# Supplementary figures and images for: Effect of a mother-baby delivery pack on institutional deliveries: A community intervention trial to address maternal mortality in rural Zambia
Source: PLoS One. 2024 Mar 11;19(3):e0296001. doi: 10.1371/journal.pone.0296001 (PMC10927137; doi:10.1371/journal.pone.0296001)

Figure 1: Recruitment algorithm

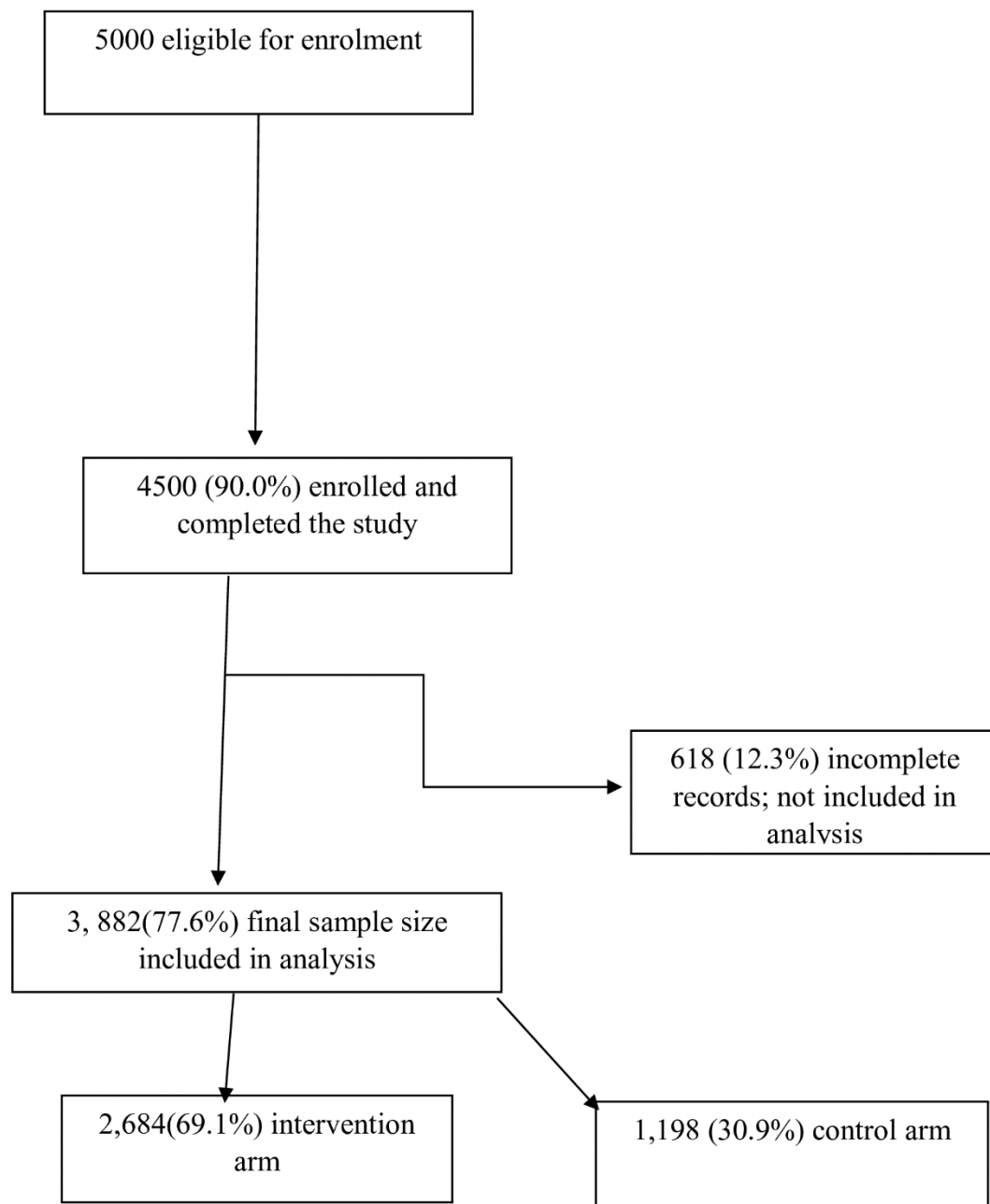

Supplement: S1 Fig — (PDF) [file pone.0296001.s001.pdf]
