# Supplementary material for: Effect of a mother-baby delivery pack on institutional deliveries: A community intervention trial to address maternal mortality in rural Zambia
Source: PLoS One. 2024 Mar 11;19(3):e0296001. doi: 10.1371/journal.pone.0296001 (PMC10927137; doi:10.1371/journal.pone.0296001)
Supplement: S1 Checklist — (PDF) [file pone.0296001.s002.pdf]

CONSORT 2010 checklist of information to include when reporting a randomised trial

**Item**	**Recommendation**	Page
Title and abstract	Identification as a randomised trial in the title	1
	Structured summary of trial design, methods, results, and conclusions (for specific guidance see CONSORT for abstracts	2
Introduction		
Background	Scientific background and explanation of rationale	4
Objectives	Specific objectives or hypotheses	5
Methods		
Trial design	Description of trial design (such as parallel, factorial) including allocation ratio	6
Participants	Enrolment and recruitment	7
	Eligibility criteria for participants	
	Describe the setting and locations where the data were collected	
Interventions	The interventions for each group with sufficient details to allow replication, including how and when they were actually administered	7
Outcomes	Completely defined pre-specified primary and secondary outcome measures, including how and when they were assessed	7
	Any changes to trial outcomes after the trial commenced, with reasons	
Study size	How sample size was determined	8
Statistical methods	Statistical methods used to compare groups for primary and secondary outcomes	9
	If applicable, explain how loss to follow-up was addressed	
Results		
Participant flow (a diagram is strongly recommended)	For each group, the numbers of participants who were randomly assigned, received intended treatment, and were analysed for the primary outcome	11
	For each group, losses and exclusions after randomisation, together with reasons	
Baseline data	Table showing baseline demographic and clinical characteristics for each group	11
Outcomes and estimation	For each primary and secondary outcome, results for each group, and the estimated effect size and its precision (such as 95% confidence interval)	11
Discussion	Interpretation consistent with results, balancing benefits and harms, and considering other relevant evidence	13
	Generalisability (external validity, applicability) of the trial findings	
Limitations	Trial limitations, addressing sources of potential bias, imprecision, and, if relevant, multiplicity of analyses	15

Conclusion		16
Funding	Sources of funding and other support (such as supply of drugs), role of funders	16
References		17
