# Supplementary material for: Effect of a mother-baby delivery pack on institutional deliveries: A community intervention trial to address maternal mortality in rural Zambia
Source: PLoS One. 2024 Mar 11;19(3):e0296001. doi: 10.1371/journal.pone.0296001 (PMC10927137; doi:10.1371/journal.pone.0296001)
Supplement: S1 File — (PDF) [file pone.0296001.s003.pdf]

A Community intervention trial for improved health facility deliveries in rural Zambia: A step towards reducing maternal mortality

INFORMED CONSENT FORM/ TEMPLATE FOR INTENDING RESEARCHERS

Background

Hello, my name is ___________________________. I am part of the research team on**: A Community intervention trial for improved health facility deliveries in rural Zambia: A step towards reducing maternal mortality**

Zambia is one of the countries with a high maternal mortality ratio (MMR) currently at 252 deaths per 100 000 live births. Limited access to institutional deliveries, poverty and socioeconomic barriers especially in rural areas are the main reasons for the high maternal mortality ratio (MMR) in developing countries. The World Health Organisation (WHO) has shown that institutional delivery by skilled birth attendants is the most important strategy to reduce maternal mortality in developing countries.

Purpose: The aim of this study is to determine the effect of provision of a non-financial incentive on institutional deliveries in form of a mother-baby delivery pack containing delivery supplies and materials provided in addition to routine ANC services this district, Monze, Zambia.

Process: You will be one of approximately 5000 pregnant women asked to participate in this study.

You will be asked information on various aspects about pregnancy, delivery as postnatal care each time you come for antenatal care at the health facility. You will also receive information about birth preparedness and newborn care. To ensure that we don't miss or forget anything during the discussion, your responses to the various questions will be recorded on the data collection sheet, but we will keep your name and signature private. Responses from the various questions will be summarised, analysed and a report written. When we write reports from your responses, we will not show what you said nor are we going to use your name or signature at any time.

Potential Benefits: You will receive no direct benefit from your participation in this discussion. However, your participation may help understand, from empirical evidence, the effect of provision of this package to pregnant women, whether it can motivate them to come to the health facility delivery. If the results are positive, we shall make recommendations to government to start providing this package to pregnant

women as they come for delivery.

Risks and Discomforts: The risks of taking part in this study are that other people will hear your responses. It is important that you do not share anything that you are not comfortable with. If you or someone in your family had a bad experience, it may be difficult or uncomfortable to remember or share it. You do not have to respond to any question unless you feel comfortable doing so. You are free to stop the discussion at any time if you need to.

.

Alternatives: You can choose not to take part in this discussion. If you decide not to take part or withdraw from this discussion, you will not suffer any penalty or lose any benefits to which you are entitled.

Participant Costs and Payments: You will not be paid to participate in this discussion. There are no costs to you for participating.

Participant's Rights: By agreeing to participate in this discussion, you do not waive any of your legal rights. Giving consent means that you have heard or read the information about this discussion and that you agree to participate. You will be given a copy of this form to keep. If you have questions or concerns at any time, you can contact the Principal Investigator,

Prof Victor Mukonka
Copperbelt University
School of Medicine
P.O. Box 91171
Ndola
Mobile: 260-977-844754,
email: vmukonka@gmail.com,

or any of the staff from the TDRC Ethics Committee on +260-21-2-61-5444

Right to Refuse or Withdraw: Taking part in this discussion is voluntary. You have the right to refuse to take part. If you decide to be in this discussion, and then change your mind, you can withdraw from it at any time and to skip questions you may deem personal or otherwise without any repercussions. Your participation is voluntary. If you choose to take part, you have the right to stop the discussion at any time.

Confidentiality: The results of this study will be kept strictly confidential, and used only for research purposes. Your identity will be concealed in as far as the law allows. Your name will not appear anywhere on the coded forms with the information. Paper and computer records will be kept under lock and key and with password protection respectively. The interviewer has discussed this information with me and offered to answer my questions. For any further questions, I may contact the Chairperson, ERES Converge on the following details____________________

STATEMENT OF CONSENT/ASSENT

.... has described to me what is going to be done, the risks, the benefits involved and my rights regarding this study. I understand that my decision to participate in this

study will not alter my usual medical care. In the use of this information, my identity will be concealed. I am aware that I may withdraw at anytime. I understand that by signing this form, I do not waive any of my legal rights but merely indicate that I have been informed about the research study in which I am voluntarily agreeing to participate. A copy of this form will be provided to me.

Name:…………………………Signature of participant ……………………..Age..……………
Date (DD/MM/YY)……………………………

Name of Witness……………………………….. Signature of Witness…………………………
Date (DD/MM/YY)…………………………………………….

Name…………………………Signature of parent or guardian for minors ……………………
Date(DD/MM/YY)…………………………

Name……………………………………..Signature of Interviewer ………………………….Date (DD/MM/YY)………………………...

If you have any further questions please contact the TDRC Ethics Committee

Telephone: 260-21-2-61-5444

By signing below you are agreeing to participate in the discussion which indicates that you have read this consent form (or have had it read to you), that your questions have been answered to your satisfaction, and that you voluntarily agree to participate in this research study. You may keep a copy of this for your records.

Signature or thumb print: ____

Date: ____

Signature of Impartial Witness: ______________________________________

Date: ____
