# Supplementary material for: Effect of a mother-baby delivery pack on institutional deliveries: A community intervention trial to address maternal mortality in rural Zambia
Source: PLoS One. 2024 Mar 11;19(3):e0296001. doi: 10.1371/journal.pone.0296001 (PMC10927137; doi:10.1371/journal.pone.0296001)
Supplement: S4 File — (PDF) [file pone.0296001.s006.pdf]

Research Proposal for PhD in Public Health

Improving Institutional Deliveries in Rural Zambia

Dr. Victor M Mukonka – PhD Student
Stage of study 2nd year

Supervisors
Dr Patricia Fitzpatrick – Principal Supervisor
Professor Fionnuala McAuliffe – Co Supervisor

Doctoral Studies Panel
Professor Patrick Wall
Dr Mary Codd
Dr Phillip Thuma

Submitted to
University College Dublin
School Public Health Physiotherapy and Population Science

Tropical Disease Research Centre (TDRC) Ethics Committee
Ndola, Zambia

11th June, 2013

CHAPTER 1

1.0 INTRODUCTION

Zambia is among the countries with the highest maternal mortality ratio in the world. It has been estimated that 591 women out of every 100,000 live births die from pregnancy related complications according to Zambia Demographic and Health Survey (ZDHS, 2007). Studies have shown that most deaths occur amongst the poorest in Zambia and particularly in the rural parts of the country.

In order to meet the Millennium Development Goal target of a Maternal Mortality Ratio (MMR) of approximately 162 deaths per 100,000 live births, Zambia would need a further reduction of 429 deaths per 100,000 live births by the year 2015.

While the country has recorded sustained high levels of antenatal care attendance, there has been poor progress in making skilled birth attendance available to all pregnant women as well as deliveries at health facilities. Many pregnant women still deliver at home, especially those in rural areas, with low education status and poor households.

According to the 1998 Zambia study on Maternal Mortality, the major causes of maternal deaths include excessive bleeding, infection, unsafe abortion, hypertension and obstructed labour. These direct causes account for fifty per cent of deaths. Contributing factors include delays in accessing health care at community and health care facility levels. Most maternal deaths occur at home or on the way to the health facility indicating that community delay is the largest contributor to maternal deaths.

Key Statistics

- Neonatal Mortality Rate: 34 per 1,000 live births
- Infant Mortality Rate: 70 per 1,000 live births
- Neonatal Mortality as a percentage of under-5 morality: 25%
- Maternal Mortality Rate: 591 per 100,000 live births
- Births are attended by a skilled health worker: 47%
- Women who do not receive any postnatal care: 51%
- Midwives availability: 5 per 1,000 live births
- Lifetime risk of maternal death: 1 in 38
- Intrapartum stillbirth rate: 12 per 1,000 births

*Source: UNICEF, UNFPA (*United Nations Population Fund*)*

In addition, most women die due to poor access to professional care. When complications occur, women do not get timely and skilled care near where they live. Distance leading to poor access to professional care also indirectly contributes to the high maternal deaths in Zambia.

Main Causes of Maternal Mortality

Therefore, the main underlying causes of maternal mortality can be grouped into the framework of the 3 delays; delay in seeking medical care (decision at home), delay in getting to health facility (transport issues) and delay in receiving appropriate medical care once the clients reach health facilities (delay of treatment at facility). The most common socio-economic barriers to institutional deliveries relate to the first 2 types of delays, leading to maternal mortality(Thaddeus and Maine, 1994).

Other compounding factors include demand by service providers at the health facility requesting mothers to bring commodities for delivery such as gloves, disinfectant (Jik), napkins, baby soap and new baby clothes. Women who are unable to afford these requirements feel embarrassed and avoid going to health facilities (Soyoola and Banda, 2010). In rural areas like Monze, some mothers use pieces of any clothing material as napkins. This causes women to shun going to the health facility when in labour because they feel embarrassed. They also fear being reprimanded by the service providers. Some women deliver at home because they do not have transport money to get back home after delivery.

Scientifically proven, evidence-based interventions to address the high maternal mortality are well known but the country experiences challenges on how to deliver them. Zambia has very high antenatal care (ANC) coverage of 93% of which 40% is attributed to an outreach programme and yet institutional deliveries are unacceptably low at 48% (ZDHS, 2007). Monze district is one of the rural districts with a low rate of skilled facility deliveries (35%). Despite its antenatal care coverage being high at 86% and many communities residing within 10km from the rural health centres (RHCs), institutional deliveries in the district remain low according to the Health Management Information System (HMIS, 2012).

Although studies are clear on the fact that institutional deliveries are low in rural Zambia, the reasons for this situation are not clear; as a result of the foregoing several questions can be raised, for example: 1) why are the majority (over 50%) of the pregnant women in rural areas of Monze not using health facilities for deliveries, and 2) what factors influence these women's choices in deciding the place of delivery. Understanding of the physical barriers to access, community perceptions and information on the factors is important for the design of interventions to address the maternal problem, and to ultimately reduce maternal mortality in Zambia; in addition, these interventions will contribute to the country's attainment of the millennium development goal number five (MDG 5).

1.1 RATIONALE

The current study will accord the country an opportunity to understand and provide in- country solutions to some of the most pertinent problems affecting poor women in rural Zambia. It will also provide an opportunity to explore other options in terms of an integrated approach to maternal health care.

HYPOTHESIS

The provision of education and a non-financial incentive in the form of a mother-baby delivery pack with key items mothers require for institutional delivery will increase the numbers of women delivered by skilled birth attendants at health facility, with subsequent reduction in maternal and neonatal mortality and morbidity.

The study will focus on **increasing the number of institutional deliveries as a key intervention for reducing maternal mortality**, based on the hypothesis that encouraging mothers to be delivered by skilled birth attendants at professionally managed health facilities will reduce the likelihood of maternal and neonatal mortality and morbidity.

1.1.1 Justification of Mother-Baby Delivery Pack

The high poverty levels in the country contribute to reduced access to health facilities, up to 59 % of Zambians are living below the poverty datum line as reported in the National Health Policy (NHP, 2012). The **non-financial incentives**, through provision of a "mother-baby delivery pack" containing basic hygienic delivery supplies to pregnant women who choose to

give birth at a health facility, will increase the number of institutional deliveries in the rural areas of Zambia. Such incentives would address some of the **hidden costs** of institutional deliveries and prevent embarrassment by women who cannot afford to purchase basic supplies requested by most rural health facilities at present.

Furthermore, the opportunity to get free supplies at health facilities aimed at making delivery and the first days of the baby more comfortable might increase the perceived quality of the institutional delivery experience, and help pregnant women overcome the willingness to continue with the tradition of home-deliveries.

Due to the reported lack of essential supplies, most health facilities request expectant mothers to bring their own supplies for delivery. The supplies demanded include gloves, napkins and baby soap. Embarrassment of not being able to afford these basic requirements presents another relevant barrier to seeking professional care at health facilities.

In 2010, the United Kingdom, Department for International Development (DFID), commissioned Mobilising Access to Maternal Health Services in Zambia programme (MAMaZ) to conduct a rapid qualitative assessment of households and community level barriers to timely utilisation of maternal and newborn health services in six districts of Zambia namely Choma, Kaoma, Mongu, Chama, Mkushi and Serenje in Southern, Western, Eastern and Central provinces respectively.

The following were the findings as regards to hidden costs of institutional deliveries:

1.1.2 Constraints to Institutional Delivery in Choma – July 2010

According to the report on rapid qualitative assessment of maternal and newborn demand side barriers done in Choma, some of the responses women gave for not delivering at a health facility included the following:-

"The clinic always wants us to go with new things, and most of us don't have new things so we don't want to go and get embarrassed."

"Some people are so poor they don't have anything to wear even for the child, so they don't want to go and embarrass themselves."

"We do not want to be embarrassed because we have not prepared anything for the baby."

"Some are afraid because they don't have clothes for the baby and the nurses will ask so if you don't have anything it is better to give birth at home."

"Women here don't go to the clinic to deliver because they know they will be required to bring some things. They ask for two new underwear and you know women in the village don't have such things, we walk around without underwear" (Soyoola and Banda, 2010).

1.1.3 Financial access barriers in Kaoma – April 2011

The insistence from most health centres that the women/family must purchase new clothes for the baby, gloves, soap and a new wrapper *(Chitenge)* for the mother for delivery at the clinic where these cannot be afforded by the family can be problematic; in this case the women will often refuse to deliver at the clinic because they fear feeling embarrassed when other mothers at the health centre have the new clothes (Surridge and Mate, 2011).

1.1.4 Chama – June 2011

According to the report, women prefer to have their babies at home rather than show up in the health facilities without the items required for delivery at the health facility because they do not want to be embarrassed and also punished or scolded by the health workers. The perspectives of members of the community were that women were required to prepare a baby shawl, a baby blanket and three wrappers costing K 105,000 (21USD) in total which is unaffordable by many community members. The report raises the need to address issues of requirements for health facility delivery and baby items (Soyoola and Ruth, 2011).

Similar findings were observed in Mongu, Mkushi and Serenje Districts where mothers raised the inability to afford required items for the baby (nappies, blanket, clothing), for the mother (new chitenge, clean underwear) and for delivery (disinfectant (Jik), gloves) as barriers to institutional deliveries.

During my work in the Ministry of Health as the Director of Technical Support Services, through my effort, the Ministry sourced financial support from the World Bank under Results Based Financing (RBF) and decided together with the District Health Office in Katete District, to pilot on a small scale an incentive scheme to increase the percentage of pregnant women delivering in health facilities. A set of demand and supply side performance-based financing mechanisms were piloted and included the provision of some basic baby requirements such as napkins, safety pins, soap and Petroleum jelly (Vaseline gel). The pack called Mama Kit was given to all new mothers who went to deliver at the health centre. Gifts were given to the traditional birth attendants for each set of five pregnant women she managed to encourage to deliver at a health facility, food was provided for antenatal mothers and 285USD given to the best performing health centre. Although the pilot scheme was not a research or a scientific community intervention trial and lacked methodology and stringent monitoring measures, we were able to demonstrate an increase in institutional deliveries overall from 55% to 85% between 2003 and 2006 in the district (RBF, 2011).

Similarly, the Ministry of Health supported a mission hospital in Chavuma District, in the North-western part of Zambia with demand side financing which included provision of some baby requirements, construction of delivery waiting shelters for mothers who must travel long distances to the facility, family planning and other educational opportunities availed to the mothers. Consequently, the district became one of the top in both facility deliveries and deliveries assisted by skilled birth attendant in Zambia, outperforming many urban districts (RBF, 2011).

The content of the Mother-baby delivery pack has been developed based on basic items needed for delivery. Recognising that malaria is a major cause of morbidity and mortality in Zambia, an insecticide treated mosquito net has been included in the pack. The mother-baby delivery pack has been costed at 20 Euro each and contains the following items for the mentioned categories below;

Baby – Two pairs of napkins, water proof pant, vest, hat, soap, petroleum jelly (Vaseline gel) and baby blanket

Mother - Wrapper (*chitenge)* for carrying the baby on the back and an insecticide treated Mosquito net (ITN) for malaria prevention, both for the mother and baby.

Delivery – Pair of gloves, disinfectant (Jik)

Taking cognisance of the fact that the challenges of distance pose one of the major barriers to institutional deliveries and will take a long time to be resolved as it will require huge investments to construct roads, improve public transport, procure ambulances for referrals, construction of new health facilities and being aware that 50% of the households in rural areas of the country are less than 5km from the health facility, some communities have local transport arrangements such as ox-carts and bicycles (NHSP, 2011). The provision of non-financial incentives in the form of mother-baby delivery pack provides a low technology, cheaper, easier to replicate and scale up intervention. It addresses one of the barriers to institutional deliveries. Considering that 86% of the mothers attend at least one antenatal care clinic during pregnancy in Monze, strengthening awareness of the importance of delivering at a health facility by skilled birth attendants, knowledge of danger signs in pregnancy and helping mothers prepare for the delivery are some of the key activities to be undertaken prior to provision of the mother-baby delivery pack at the health facility (HMIS, 2012).

In the middle, a mother who has delivered accompanied by grandmother with a blue headscarf holding the baby and the aunt being transported in an ox-driven cart after delivery at a health facility

If the non-financial incentive in the form of mother-baby delivery pack shows effect, this intervention has the potential of being developed into a delivery pack which could be supplied to rural health facilities on a regular basis similar to the current Rural Health Centre drug kit. This would make a huge difference for the majority of poor people living in rural parts of Zambia who cannot afford the hidden costs and ultimately lead to increased institutional deliveries. The World Health Organisation both at global and country level has shown interest and commitment to the study (see letters in annex).

This study will contribute to a better understanding of factors and barriers associated with institutional and home deliveries. The information obtained will be important for use in policy formulation and development of in-country evidence-based strategies and interventions for promoting institutional deliveries. The study will also assist in providing information for developing a better delivery pack and improved guidelines regarding incentives to increase institutional deliveries.

Through provision of non-financial incentive as a way to encourage women to deliver at health facilities by skilled birth attendants, it is anticipated that the study will ultimately lead to a reduction in maternal mortality particularly in poverty- stricken rural parts of Zambia.

Furthermore, by publishing results of this study in Public Health journals, the study will contribute to the regional and global pool of knowledge on factors and barriers associated with institutional and home deliveries and innovative ways of promoting institutional deliveries, through provision of non-financial incentives.

1.2 Research questions

To determine the factors associated with place of delivery and the effect of provision of a non-financial incentive in order to contribute to the reduction of maternal mortality and complications associated with child birth in Monze District, a rural area of Southern Province of Zambia.

The specific objectives of this research are as follows:

(1) To establish factors associated with institutional and home deliveries in rural areas of Monze District include; barriers to timely ultilisation of health care services, socio-economic, cultural, traditional practices and beliefs, gender inequalities, knowledge and attitudes, distance, financial barriers, staff attitude and behaviour, knowledge of maternal danger signs.

(2) To determine the extent to which non-financial incentives in the form of mother-baby delivery pack accompanied with health education and information leaflet contribute to increasing the institutional deliveries in rural areas of Monze District over a period of one year, from 1st January to 31st December 2014.

(3) To determine husbands' perceptions, role and involvement regarding place of delivery in Monze district, Zambia.

The study will be conducted in three phases. The first phase will be a community observational descriptive cross-sectional study of pregnant women and women who have delivered in the past 10 years in Monze district that will aim to establish the factors associated with institutional and home deliveries. It will be done within six (6) weeks starting on 20th November 2013 in Monze district. The second phase of the study will be an experimental prospective comparative community intervention trial with two arms: the control group will receive routine standard health services provided at health facilities in the district; the intervention arm will receive non-financial incentives in the form of a mother-baby delivery pack which will contain a pair of delivery gloves, baby napkins, bottle of petroleum jelly (Vaseline gel), baby soap, baby vest, a mother's wrapper (*chitenge)* for carrying the baby on the back, and an insecticide treated mosquito net (ITN). The inclusion of a *chitenge* is meant to serve as an incentive for the mother dressing and carrying the baby on the back and an ITN for malaria prevention for both mother and baby. It will be conducted over one year, starting on 1st January 2014. The third study will be a qualitative study with the main aim of gaining understanding of the husbands' role and involvement in the women's decisions regarding the place of delivery in rural Monze, Zambia. The study will be done in November and December, 2014.

CHAPTER 2

2.0 LITERATURE REVIEW

2.1. Literature Search Strategy

The literature search was conducted using the PICO system to identify key terms. The PICO system has four main components namely patient or population, intervention or exposure, comparison and outcomes. Using the system, low institutional deliveries leading to high maternal mortality in rural areas of Monze was identified as the major problem and the intervention proposed for the study being non-financial incentives in the form of mother-baby delivery pack. The comparison would be between the intervention and control arms with outcome measure being the number of institutional deliveries.

The databases accessed included government reports looking at the health policies, national health strategic plans, the national maternal, newborn and child health road map, health management information system and joint annual and programme reviews. Other data bases reviewed were United Nations agencies reports from WHO, UNICEF, UNFPA and World Bank on maternal, newborn and child health. The reports reviewed included amongst others; trends in maternal mortality 1990 – 2010, 2012 report, measuring maternal mortality, women deliver, countdown to 2015 for maternal, newborn and child survival.

The literature search strategy included accessing data bases using Google Scholar and PubMed search for systematic reviews and peer reviewed articles and publications on home and institutional deliveries as it relates to maternal mortality. Subject headings and key words such as institutional deliveries, maternal mortality and factors associated with home deliveries, access to institutional deliveries, barriers to institutional deliveries, incentives for increasing institutional deliveries were used as search terms. The search strategies involved the use of the following different operator combinations of AND/OR; maternal mortality AND institutional deliveries, home deliveries AND/OR maternal mortality, institutional deliveries AND barriers, factors AND institutional deliveries, utilization AND/OR maternal health services, home deliveries AND institutional deliveries, determinants AND home deliveries.The searches were limited to English systematic reviews, peer reviewed articles and for a period covering from 2000 to 2013.

2.2. Country Profile

2.2.1. *Geographical location*

Zambia is a landlocked country in sub-Sahara, South Central Africa, between 8° and 18°south latitudes and 22°and 34° east longitudes. It has a total surface area of about 752,614 square km and shares boundaries with eight neighbouring countries namely the Democratic Republic of Congo to the north, Tanzania to the north-east, Malawi to the east, Mozambique, Zimbabwe, Botswana and Namibia to the south, and Angola to the west. The capital city is Lusaka located in the south-central part of the country. The official language or medium of communication is English. Its official currency is in the form of notes and coins called the Kwacha and Ngwee respectively.

Administratively, the country is divided into ten provinces, namely Central, Copperbelt, Eastern, Luapula, Lusaka, Muchinga, Northern, North-Western, Southern and Western provinces. These provinces are further subdivided into a total of one hundred and ten districts.

2.2.2. *Demographic situation and trends*

The population of Zambia has rapidly grown from about 3 million people in 1964, to 13.2 million in 2010 with the male population at 6.5 million (49.3%) and the female at 6.6 million (50.7%). Zambia's population density increased from 7.5 people per square kilometre in 1980 to 9.8 in 1990 to 13.1 in 2000 and now stands at 17.3 persons per square kilometre. The average life expectancy at birth has also increased from 40.5 years in 1998 to 51.3 years in 2010. This rapid population growth places an increasing burden on the national economy, particularly the country's capacity to keep pace with the health needs of a rapidly increasing population and its dynamics. The population residing in the rural areas stands at 7.9 million (60.5 per cent) while the urban population is 5.1 million (39.5 per cent) (CSO, 2010).

2.2.3. *Income and socio-economic Status*

Over the past five years, the country has registered consistent economic growth, averaging 6.2 per cent growth in the Gross Domestic Product (GDP) per year, and significant improvements in other key macro-economic indicators. However, these achievements have not yet significantly impacted on the socio-economic status of the majority of the population, most of who have continued to face poverty and socio-economic deprivation. The situation is further compounded by the inequities in the distribution of wealth and socio-economic infrastructure across the country, which currently favours the urban areas and adversely impacts on the provision of social services, such as health and education in the rural hard-to-reach areas. The

country is experiencing high levels of unemployment and weak socio-economic status of the population, which have implications for the health status of the population. Income inequity among the population has remained high, with the Gini Coefficient at 0.57 in 2004 (a drop from 0.66 in 1998) (NHSP, 2011).

According to the Living Conditions Monitoring Survey of 2006, 64 per cent of Zambians were classified as poor, with higher poverty levels in rural than urban areas (80 and 34 per cent, respectively).

2.2.4. *Healthcare Financing*

Government spending on health accounts for 60% of total public health sector funds. This represents 10.7% of the central government discretionary budget or 8.5% of the total national budget, which is greatly outside the Target of 15%, which was set by the Heads of States at the African Union summit in Abuja, Nigeria. As a percentage of the Gross Domestic Product (GDP), health care spending represents 5.4% to 6.6%, which translates to approximately US$ 28 per capita. Other sources of health care financing include user fees, which until the introduction of the User Fees Removal Policy for rural and peri-urban areas in 2006 represented about 4% of total health care financing. About 40% of total health expenditure comes from various cooperating partners (NHSP, 2011).

2.2.5. *Health Care Delivery System*

Health services in Zambia are delivered through three levels with the main aim of bringing the services as close to the family as possible through the Primary Health Care approach. The National level is responsible for overall coordination and management of the health sector and specifically policy formulation, strategic planning, resource mobilization and donor coordination. The provincial level which is the link between the central and district levels is responsible for performance assessment and technical support to the lower levels. The District level is responsible for implementation of health promotion, preventive, curative and rehabilitative services. Administratively, the District Health Office is responsible for coordination of provision of health services in the district. Each district has a District Hospital which provides the first level care in medicine, obstetrics & gynecology, surgery and pediatrics. Below this level, there are health centres and health posts providing both static and outreach activities. The health facilities refer difficult or complicated cases to the District Hospital. The main activities at health centre and health post level are predominantly health promotion and disease prevention. They also provide curative services. Each health centre is responsible for running key health programmes which include maternal, newborn and child health, communicable and non-communicable diseases, environmental, water and sanitation, school health and nutrition and epidemic preparedness.

2.2.6. *Delivery Structure*

a. A Health Post caters for a population of 500 households (3,500 people) in rural areas and 1,000 households (7,000 people) in urban areas within a 5 Km radius for sparsely populated areas.

b. Urban Health Centres cater for a catchment population of 30,000 to 50,000 people.

c. A Rural Health Centre caters for the catchment area within a 29 Km radius or population of 10,000 people.

d. A Level-1 Hospital caters for a population of between 80,000 and 200,000 with medical, surgical, paediatric, obstetrics and gynaecology and diagnostic services to manage referrals from health centers.

e. Level -2 Hospitals cater for a catchment population of 200,000 to 800,000 people with services in internal medicine, general surgery, paediatrics, obstetrics and gynaecology, dental, psychiatry and intensive care services. They also act as referral centres to the first level hospitals.

f. Level-3 Hospitals cater for catchment populations of 800,000 and above, and have sub-specialisations in internal medicine, surgery, paediatrics, obstetrics, gynaecology, intensive care, and psychiatry. Level-3 Hospitals also provide training and research. These hospitals act as referral centres to second level hospitals.

g. Other providers of health care includes (i) **Faith-based facilities,** owned by the various Christian Church mother bodies, under the coordination of Churches Health Association of Zambia and (ii) **Private health facilities,** for and not for profit facilities owned by private investors and Civil Society Organizations (CSOs).

Despite having the structures in place, the health sector continues to face major challenges which include high disease burden, inadequate medical staff, weak logistics management in the supply of drugs and medical supplies including delivery requirements, inadequate and inequitable distribution of health infrastructure, equipment and transport. Other challenges relate to health information systems, inadequate financing and weaknesses in the health systems governance. The health services are fragmented and unevenly distributed with rural parts of the country severely affected.

2.2.7. *Disease Burden, situation and trends*

The burden of disease in Zambia is high, and is largely influenced by the high prevalence and impact of communicable diseases, particularly malaria, HIV and AIDS, Tuberculosis (TB) and Sexually Transmitted Infections (STIs). The country is also faced with a high burden of Maternal, Neonatal and Child Health (MNCH) problems, and a growing problem of Non-Communicable Diseases (NCDs), including mental illness, cancers, sickle cell anaemia, diabetes mellitus, hypertension and heart diseases, chronic respiratory disease, blindness and refractive visual defects, and oral health problems.

2.2.8. *Maternal health care*

Maternal and child health services are provided at all levels of health care delivery system. Most of the primary health care as it relates to maternal health is carried out at health centre level including conduct of normal deliveries. All pregnancies requiring caesarean section or with complications are referred to the first level hospital. The challenges being faced are similar to those faced in other parts of the health services, and include weak referral systems and absence of emergency systems for handling obstetric, neonatal and child health emergencies, and poor logistics management of drugs, delivery supplies, family planning commodities, vaccines and equipment.

2.3. Maternal Mortality Trends in African Region

Maternal health is one of the major worldwide health challenges. Currently, the unacceptably high levels of maternal mortality are a common subject in global health and development discussions. The risk of a woman dying as a result of pregnancy or childbirth during her lifetime is about one in six in the poorest parts of the world compared with about one in 30 000 in Northern Europe. Such a discrepancy poses a huge challenge to meeting the fifth Millennium Development Goal to reduce maternal mortality by 75% between 1990 and 2015 (Ronsmans and Graham, 2006).

Although some countries have made remarkable progress, half of the maternal deaths in the world still take place in Sub-Saharan Africa where little or no progress has been made. The combined maternal mortality ratio for severe bleeding, hypertensive diseases, and infections is staggering at almost 500 deaths per 100 000 live births in sub-Saharan Africa, compared with fewer than 300 per 100 000 in south Asia, just over 100 in Latin America and the Caribbean, and 4 per 100 000 in developed nations(Ronsmans and Graham, 2006).

There is no single simple, straightforward intervention that will significantly decrease maternal mortality alone; however, there is a consensus on the importance of a strong health system, skilled delivery attendants, and women's rights for maternal health (Alvarez et al., 2009). Targeting of interventions to the most vulnerable rural populations and poor people is essential if substantial progress is to be achieved by 2015.

In the sub-Saharan Africa region, births attended by skilled health personnel and life expectancy at birth strongly correlate with maternal mortality. Gross national product (GNP) per capita and health expenditure per capita also have strong association with maternal mortality (Buor and Bream, 2004).

Maternal Mortality in Africa

According to the 2010 estimates by WHO published in 2012, 26 countries have made progress and 2 are on track towards attainment of the MDG 5. About 162,000 maternal deaths occurred in the African Region in 2010. This constituted over half (56%) of the total maternal deaths in the world that year (287,000). Countdown tracks progress in the 75 countries where more than 95% of all maternal and child deaths occur. The report has shown that both maternal and child mortality is dropping in most countdown countries but slower declines in Sub-Saharan Africa, where fertility remains higher than in other regions. Maternal mortality is higher in women living in rural areas and among poorer communities. Young adolescents face a higher risk of complications and death as a result of pregnancy than older women (Bhutta et al., 2010).

2.4. The Situation and Current Effort in Zambia

In Zambia, **maternal health still represents a major challenge**. It is estimated that eight women die every day due to pregnancy and childbirth complications in the country. Despite over 20 years of efforts in applying the Safe Motherhood Initiative (SMI), the maternal mortality ratios have been fluctuating, rising from an estimated 200 per 100,000 live births in 1992 to 729 deaths per 100,000 live births in 2001 – 2002 and 591 in 2007 (ZDHS, 2007). The MDG target set for MMR by 2015 in Zambia is 162.

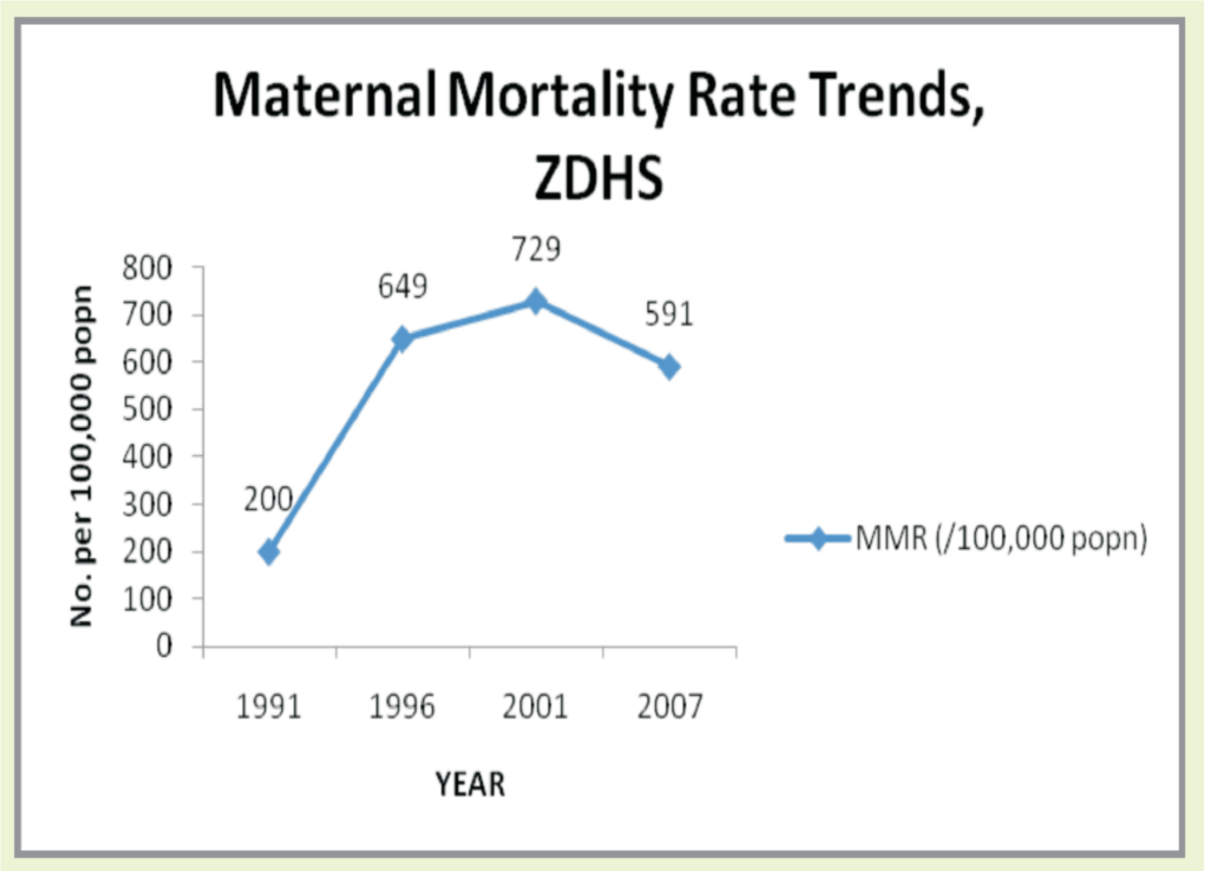

Figure 4 **Maternal Mortality Rate Trends**

With regards to child health, Zambia is still among countries with high under five mortality rate (U-5MR) in the region at 119 per 1,000 live births, despite a decrease from 168 per 1000 live births in 2001. Neonatal mortality contributes significantly (40%) to the U-5MR. Neonatal care has remained stagnated in the range of 34-37 deaths per 1000 live births over the last two to three decades as interventions in the category have been fragmented with limited health worker skills and capacity to manage newborns. The major causes of child mortality are malaria, respiratory infections, diarrhoea, malnutrition and anaemia. HIV/AIDS is increasingly contributing to morbidity and mortality in children.

Most under five deaths occur at home because they do not access health care for several reasons including long distances to health facilities, poor quality of health services and inadequate outreach services. In order to reach the Fourth MDG target of 64 per 1000 live births, there is need to ensure that life saving child survival including neonatal interventions reach communities and families where they live.

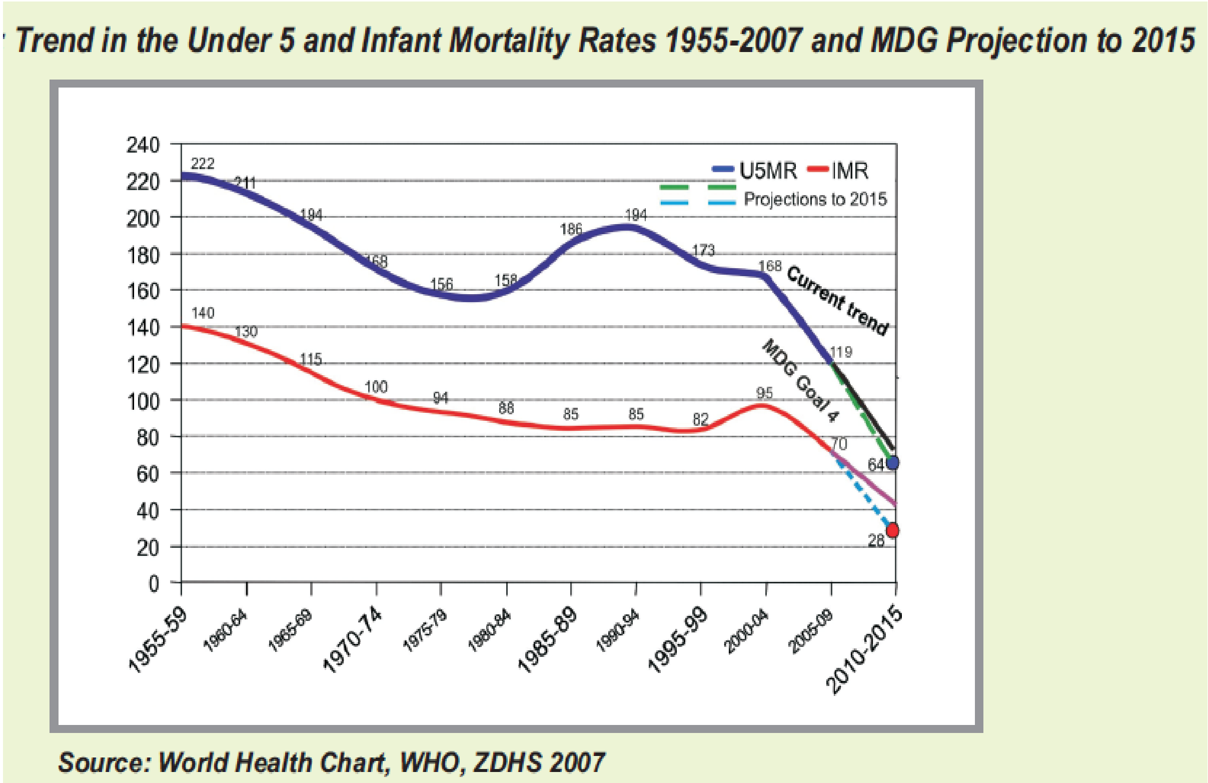

Figure 5 Trends in the Under Five and Infant Mortality Rates

Zambia has recorded sustained high levels of antenatal care attendance (in excess of 90% coverage) but without corresponding increase in institutional deliveries by skilled birth attendants. There has been poor progress in making skilled birth attendants available to all pregnant women as well as deliveries at health facilities. Institutional deliveries have constantly remained at less than 50 per cent since 2001. The Zambia Demographic and Health Survey showed the rate of institutional deliveries was 43 per cent, with a slight increase in 2007 to 48 per cent whereas antenatal care (ANC) coverage (1 visit) was 93 per cent in 2001 and 93 per cent in 2007. Of significance to note was the decrease in ANC Coverage (4 visits) from 70 per cent in 2001 to 63 per cent in 2007 (ZDHS, 2007). The study on waiting too long, low use of maternal health services done in Kalabo District of Zambia showed that the large difference between the reasonably high attendance and the lower supervised delivery is still not fully understood. ANC attendance did not influence the decision where to deliver. Only 15% of women who visited the ANC had adequate knowledge about the risk factors and danger signs of pregnancy. The study observed that women who had a high level of knowledge about risk factors of pregnancy more often delivered in a health institution and women who made their own decisions had a higher chance of delivering in a health facility (Stekelenburg et al., 2004).

According to the maternal, newborn and child health (MNCH) road map for Zambia many pregnant women still deliver at home, especially those from rural areas, with low education status, and women from poor households. Women from poor households, due to their socio-economic status and socio-cultural perceptions around pregnancy and birth, also attend fewer antenatal care visits, which tend to be of lower quality than those from urban areas as most rural health centres are poorly staffed and experience shortage of equipment and medical supplies. These disparities in use of care are also compounded by considerable difficulties in accessing health care when needed (MNCH, 2012). In the study on the influence of distance and level of care on delivery place in rural Zambia, both distance to service and quality are

important determinants of health facility use. Women and their families do make assessment of some aspects of quality and these assessments influence the distance they are willing to travel. Policy should focus on health system determinants in particular geographic and quality barriers to obstetric care (Gabrysch et al., 2011).

The main mode of transport in Zambia for poor and rural women in seeking care is by walking and usually requires being accompanied by their husbands or other family members. According to the rapid assessment done by Mobilising Access to Maternal Health Services in Zambia, distance and lack of transport to go to the health facility were major factors in accessing maternal and newborn health services. The pregnant mother had to weigh probability of the risk occurring with delivering at home against the challenges of delivering at health facility (Surridge and Mate, 2011).

Other factors that have contributed to high maternal mortality rate in Zambia apart from transport challenges includes, a weakening health system, lack of appropriate equipment and shortages of medical supplies especially linked to delivery requirements at health facilities, as well as indirect causes mainly due to HIV, malaria, tuberculosis and underlying malnutrition.

The high poverty level in the country contributes to reduced access to facilities, as well as the high prevalence of malnutrition and anaemia among pregnant women. Delivery preparedness is generally very poor in rural areas in terms of keeping ready and clean clothes for the mother and baby for use during delivery, making critical preparations such as arranging transport, identifying an institution for delivery in case of an emergency and saving money.

Despite the fact that health services at public health institutions in Zambia are free of charge, there are often **hidden costs** to institutional deliveries that are not irrelevant for the poorest households in rural areas. For instance, due to the reported lack of essential supplies, most health centers request expectant mothers to bring their own supplies for delivery, including gloves, napkins and baby soap. Embarrassment of not being able to afford this basic requirement represents another relevant barrier to seeking care at health facilities.

2.5. The Situation and Current Effort around the World

The study on barriers to the utilisation of maternal health care in rural Mali found that the significant area variation in the utilisation of maternal health services remained unexplained although transportation barriers, lack of education and influences by others in the area of residence were factors (Gage, 2007). Another study understanding the factors associated with institutional delivery service utilisation in Ethiopia found that the main factors that had an influence on health institutional delivery included education of both mother and husband, residing in the urban area and attendance at antenatal care (Shiferaw et al., 2013).

In Uganda, according to a study on increasing access to institutional deliveries using demand and supply side incentives, the main reasons for women not delivering in a health facility includes financial limitations, long distances to health facilities coupled with transport difficulties, lack of decision making power among women, inability to afford the medical supplies that are often compulsory at public health facilities, rude health workers and preference for traditional child birth attendants (Ekirapa-Kiracho et al., 2011).

According to Shiferaw et al (2013), in Ethiopia women prefer home births because they believe it is not necessary to deliver at the health facility (42%), it is not customary (36%), it is high cost (22%) or due to distance or lack of transportation (8%), whereas in Malawi, home deliveries were associated with being resident in the central region, no formal education, never

used contraceptives, poor women with large number of children and poor attendance at prenatal visits (Palamuleni, 2011).

In a study done in rural Uttar Pradesh of India on increasing institutional deliveries and access to emergency obstetric care services, it was observed that birth preparedness including saving money, arranging transportation and identification of health facility to deliver from where an important component of safe delivery (Varma et al., 2010).

In Malawi, in a study on determinants of non-institutional deliveries, it was observed that poverty was a major factor for non use of health facilities during delivery. Other factors included poor accessibility in terms of distance, inadequate transport, infrastructure and culture (Palamuleni, 2011). In terms of cultural factors, a number of women have to seek permission from husbands or husband's relatives to go to the health facility and shy away from health facilities that are staffed by male midwives. According to (Varma et al., 2010) the key reasons for the continued prevalence of home deliveries assisted by traditional birth attendants (TBAs) are the family's trust in the ability of TBAs, their desire to follow family customs and rituals regarding birthing and the easy accessibility of TBAs within the community.

According to a review study "still too far to walk; literature review of the determinants of delivery service use", most studies of the determinants of skilled attendance have concentrated on sociocultural and economic accessibility and neglected perceived benefits/need, physical accessibility, community determinants such as community attitudes and norms concerning childbirth. Gaps exists on information regarding perceived benefit/need, physical accessibility and measuring complications (Gabrysch and Campbell, 2009).

In order to address the low institutional deliveries, a number of countries have been trying various interventions. In Cambodia according to a case study done in three rural health districts, using targeted vouchers and health equity funds to improve access to skilled birth attendants for poor women, facility deliveries increased sharply from 16.3% of the expected number of births in 2006 to 44.9% in 2008 after the introduction of voucher and health equity funds schemes (Ir et al., 2010). The increase was much more substantial than in comparable districts over the same period without health equity fund schemes. The vouchers covered free services at the health centre for three antenatal care visits, delivery and one postnatal care visit and transportation costs for five round trips between home and the health centre and for referrals from health centre to hospital in case of complications. The study concludes by indicating that vouchers and health equity fund schemes if carefully designed and implemented have a strong potential for reducing financial barriers and hence improving access to skilled birth attendants for poor women (Ir et al., 2010).

Addressing maternal health care through financial incentives, the experience of the Janani Suraksha Yojana (JSY) programme in India showed an increase in skilled birth attendance, antenatal and postnatal care. The gains in institutionalisation of deliveries are far greater than those of antenatal and postnatal care, indicating the limited role of the JSY in comprehensively addressing the maternal care needs (Gopalan and Durairaj, 2012). In the Uganda study on increasing access to institutional deliveries using demand and supply side incentive, the authors concluded that the use of demand and supply side incentives through community financial incentives, loan/insurance schemes and maternity waiting homes seem promising (Ekirapa-Kiracho et al., 2011).

According to a study done in rural Uttar Pradesh of India on increasing institutional deliveries and access to emergency obstetric care services, when financial incentives were introduced,

74% of the women reported financial incentives subsidised their out of pocket expenses (Varma et al., 2010). The other intervention introduced was community-based female volunteers called Accredited Social Health Activists; these women are given a performance-linked fee for a number of women they motivate for ANC and institutional delivery. (Varma et al., 2010) concludes by indicating that the scheme is promising but still needs closer examination to assess its performance.

CHAPTER 3

3.0 METHODOLOGY

3.1. Study site

The study will be carried out in Monze district in Southern Province of Zambia. It is situated 200km South of Lusaka, the capital City, and 300km North of Livingstone Town. The District covers an area of 6,687 kilometer square. The inhabitants of Monze are mainly the Batonga people. The Batonga have a very rich traditional culture and great "Bulemu" for other people. Bulemu means respect. They show deep respect to one another and to all other people. Their main attitude to life is `peace at all costs`. The Batonga people will never push themselves into the limelight or try to dominate others. They are a humble, quiet and reserved tribe. The Batonga people have a paramount chief called Chief Monze, who was recognised by Queen Elizabeth II of Great Britain in pre-colonial times when she first visited the country. The paramount chief is assisted by headmen. There are over 300 villages in Monze, each headed by a headman. The people of Monze live in very scattered and isolated communities. They are mainly peasant farmers involved in small-scale growing of crops and rearing of cattle, goats and chickens.

Monze district has a population of 203,038, a General Hospital and 26 health centres. All the health centres provide maternal and child health services and conduct deliveries except 5 which do not conduct deliveries, the 3 urban health centres namely Monze urban, Manungu, ZCA and 2 small rural health centres namely Charles Lwanga and St Mary's. Monze General Hospital serve as the main referral hospital for the whole district, perform caesarean sections and deals with all complicated cases referred from the health centres.

In 2012, the number of expected pregnancies was 10,964 and expected deliveries 10,558. The antenatal coverage was 86% and institutional deliveries 35%. In terms of the road infrastructure, Monze has mainly gravel roads. However, maintenance is lacking resulting in a state of disrepair and making traveling by road very difficult. Communication between the District Health Office and the Health Centres is facilitated by the use of communication radios, land phones and Mobile Cell Phones. The weather pattern consists of the wet season from November to March, the cold season from April to July and the hot season from August to October.

3.2. Study Type

The study will be conducted in three phases. The first phase will be a community observational descriptive cross-sectional study of pregnant women and women who have delivered within the last 10 years in Monze district that will aim to establish the factors associated with institutional and home deliveries in rural areas of Monze District. The second

phase of the study will be an experimental prospective comparative community intervention trial of non-financial incentives in the form of mother-baby delivery pack, with two study arms: the control and the intervention groups. The third phase will be a qualitative study exploring husbands' perceptions regarding the place of delivery, determining their role and involvement and identifying factors influencing their decision regarding the place of delivery. The detailed methodology for each study is described in the following chapters.

CHAPTER 4

4.0 CROSS-SECTIONAL STUDY - (PHASE 1)

4.1. Aim

The main aim of the observational descriptive cross-sectional study will be to gain an insightful understanding of the socio-economic factors preventing institutional deliveries in rural Zambia (i.e. barriers to timely utilisation of health services, culture, traditional practices and beliefs, gender inequalities, knowledge and attitudes, distance, financial barriers, staff attitude and behavior, knowledge of maternal danger signs). The study will be conducted within six (6) weeks starting on 20th November 2013 in Monze district.

4.2. Objectives

To establish factors associated with institutional and home deliveries in rural areas of Monze District, specifically;

- Identify the factors which influence the choice of place of delivery by pregnant women in rural parts of Monze District including health facility utilisation.

- Establish the socio-economic, cultural and logistical barriers influencing utilisation of health facilities for deliveries in rural parts of Monze District.

- Explore knowledge, attitudes and practices contributing to low institutional deliveries in Monze District.

- Document and provide evidence to policy makers on barriers to institutional deliveries for better and effective design of interventions to increase institutional deliveries in rural parts of Zambia and ultimately reduce maternal mortality and morbidity.

4.3. Methodology

4.3.1. *Study site and population*

The study will be carried out in Monze District in Southern Province. The district has a population of 203,038 and 26 health facilities. The study population will be pregnant women and women who have delivered within the last 10 years in Monze district. The total estimated number of pregnancies in Monze in 2012 was 10,964.

4.3.2. *Study design*

The study will be an observational descriptive cross-sectional survey of pregnant women and women who have delivered within the last 10 years in Monze district that will aim to establish the factors associated with institutional and home deliveries in the rural areas of Monze District. The study will be conducted within six (6) weeks starting on 20th November 2013.

4.3.3. *Sample Size determination*

The following would have to be set in order to derive a valid sample size, based on the assumption that the research aims at estimating a population proportion with a characteristic of interest.

- Select a confidence level
- Specify a margin of error (e.g $\pm 5\%, \pm 10\%$ etc. A margin of error of 5% and below is very good, 10% would be considered good and below 20% could be acceptable. (the smaller the margin of error the bigger the sample size)
- Decide on a method of sampling
- Obtain the population Size
- Assume a response rate

(i) Number of domains=1

Since Monze District is the only study district and that these results will be reported at district level then the calculation of sample size will consider only one domain.

(ii) Confidence level

The chosen confidence level is 95% (this is recommended). The z-value is 1.96

(iii) ***Margin of error***

The margin of error 0.05 is chosen. A margin of error of 5% and below is very good, 10% would be considered good while below 20% could be acceptable.

(iv) Sampling Method

The sampling method will be two stage cluster sampling and therefore the design effect of 2 is chosen as recommended.

(v) Population Size

The population size of Monze District is 203,038.

(vi) Response Rate

A response rate of 85% is assumed. This is based on the past experience where household response rate in rural areas has been usually above 90%, see below the data from the Zambia Demographic & Health Surveys and the Sexual Behaviour Surveys done in the past. Recently in November 2012, Zambia, with technical support from WHO conducted a national post-measles immunisation campaign coverage survey which had a 99.7% response rate.

ZDHS response rates

	**Household**	**Women**	**Men**
1996	98.9	96.7	90.1
2001	98.2	96.4	83.7
2007	97.8	96.5	91.0

Sexual Behaviour Survey response rates

	**Household**	**Women**	**Men**
1998	97	95	92
2000	92	88	85

2003	93	87	85
2005	93	88	86

The sample size has been calculated in steps as follows:
(Reference – Allen Gower & Karen Kelly, Social Survey Methods, Statistics Division, Canada – 1993)

Step 1

$n_1 = \frac{z^2 pq}{d^2}$ $n_1 = \frac{1.96^2 * (0.5 * 0.5)}{0.005^2}$ =**384.16**

Where *p* is an estimate of the proportion of the population that has the characteristic of interest or the probability of success and q = 1-p. The safest choice is when p=0.5 when there is no estimate from any study yet.
z = (1.645 for a 90 % confidence interval)
z = (1.96 for a 95 % confidence interval)
z = (2.575 for a 99 % confidence interval)

d = the specified margin of error which is 0.05.

Step 2

Calculate the modified sample size (n_2) to take into account the population size:
$n_2 = n_1 \frac{N}{N + n_1} = 384.16 * \frac{203,038}{203,038 + 384.16}$ =**384.1581**

Where, N is the size of the survey population =203,038 in this case.

Step 3

An adjustment for the design effect is made using the formula:
$n_3 = Bn_2$ = 384.41581*2=**768.3162**

Where B (design effect) = 1 for simple random sampling design
B<2 for stratified sampling design
B>1 for cluster multistage sampling designs
The method of sampling will be stratified cluster sampling and a design effect of 2 is chosen.

Step 4

Adjusting for non-response
$n = \frac{n_3}{r}$ =768.3162/0.85=**903.9014**
Where r is the expected response rate

These calculations are done for a single domain and a sample size of about **905** cases would provide reliable estimates for the study.

The sample size of 905 is the number of household interviews which will be achieved. All eligible persons (all pregnant women and women who have delivered within the last 10 years in Monze district) found in the selected household will be interviewed. Non responding households or households with no eligible respondent will not be replaced. The calculation of the sample size factored in a 15% non response.

4.3.4. *Sampling Framework*

The sampling frame for the cross-sectional study will be a list of all enumeration areas (EAs) for Monze District (these are statistical demarcations usually taken as primary sampling units) available with Central Statistical Office. The sample will then be selected in two stages the first stage will select the enumeration areas or clusters and the second stage will select households. In the selected households the interviews will be conducted with eligible respondents (all pregnant women and women who have delivered within the last 10 years in Monze district).

(i) First Stage Sampling

Primary sampling units i.e. Enumeration areas will be selected from the 2010 census frame in the first stage. The required number of enumeration areas is 34. This is based on the total number of households required, which is 905. Learning from experiences with CSO surveys done in the past in Zambia amongst them the Zambia demographic and health surveys, Sexual behaviour surveys, 25 households selected from a selected cluster is considered the optimum.

The structure of the census frame to be used to select the primary sampling units is as described below:-

Administratively, Zambia is divided into ten provinces. Each province is in turn subdivided into districts. For statistical purposes each district is subdivided into Census Supervisory Areas (CSAs) and these are in turn subdivided into Enumeration Areas (EAs). CSAs are grouped in wards, wards in constituencies, constituencies in districts and districts in provinces. The listing of EAs has information on number of households and the population. The number of households is used as a measure of size for selecting primary sampling units
(PSU).

The EAs are also grouped into urban and rural categories. Stratification by rural and urban grouping of EAs is necessary since this study is focusing on the rural part of Monze. In total there are 354 enumeration areas for Monze district according to the 2010 census of population and housing; 292 are rural and 62 are urban EAs.

(ii) Second Stage Sampling

In each of the 34 selected EA, the number of households will be listed and numbered. 25 Sample households will be selected systematically using a pre-determined interval.

(iii)Listing households

The selection of households will be done after a listing of all households in the selected EAs. Each household is given a unique sampling serial number.

(iv) Selection of households

A Systematic Sampling Procedure will be used because it is simple, involves getting the random start from the table of random numbers (The first number that falls between 1 and N (which is the total number of households listed is the random start)

Select household by adding k (sampling interval) to the serial number of each selected household until you achieve your "n" (25 households). From all the clusters a total of 905 households will be achieved.

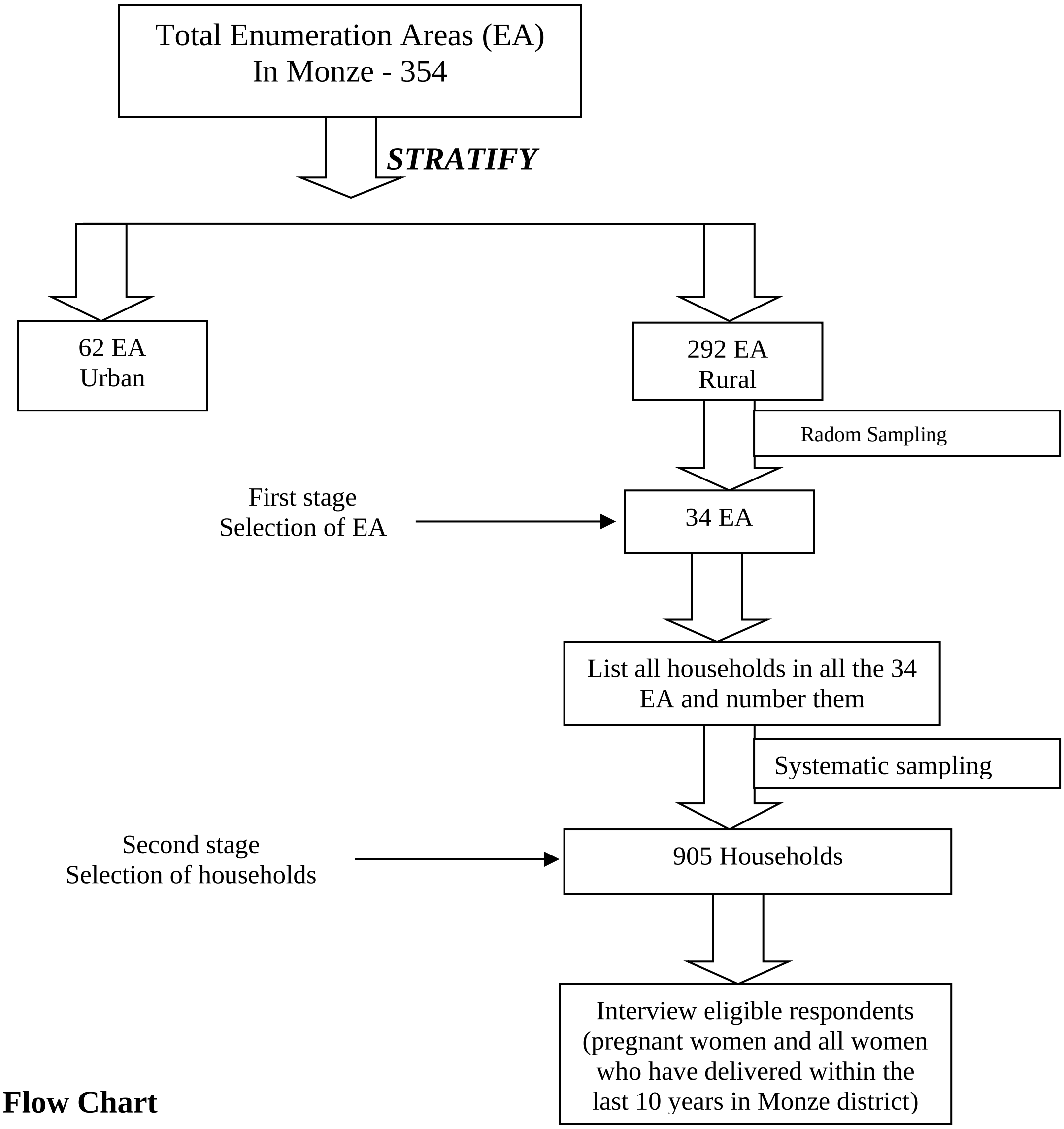

Figure 6 Flow Chart

4.3.5. *Study Instrument*

Interviewer administered structured questionnaire will be used by data collectors to collect primary data. The questionnaire and format for survey interviews have been developed based on the research question "Why is the majority (over 50%) of the pregnant women in rural areas of Monze District in Zambia not using health facilities for deliveries? What factors influence women's choices in deciding the place of delivery".

In developing the questionnaire different sources of established validated data collection instruments have been used, modified to suit the study and consideration has been made to ensure questions were not leading, unclear, long, contradicting, irrelevant and poorly worded. Similar questions have been put together, with inoffensive questions first. The questionnaire

provide for introduction, instructions to follow with emphasis on confidentiality, anonymity and at the end thanking the respondent for taking part.

The different sources of established validated data collection instruments modified and used to develop the questionnaire include the following;

- Zambia Demographic and Health Survey instruments used for the questions on socio-economic, maternal health seeking behavior, delivery preparedness, antenatal care attendance, barriers to institutional deliveries (Zambia Demographic and Health Survey 2007 Report, Central Statistical Office, Lusaka, Zambia).

- Malaria Indicator Survey for questions on sources of information on maternal health care services (National Malaria Indicator Survey 2010 Report, Ministry of Health, Zambia).

- World Health Organisation Instruments on delivery preparedness, danger signs in pregnancy (Every Woman Every Child Task Force on Checklists, WHO Report, 2012).

- Harvard University Survey Instruments on demographic information (Professor Nava Ashraf, Baseline Survey, Harvard Business School, Harvard University, 2009).

Data Dictionary

The data dictionary has been developed using the excel spreadsheets defining all the variables in the data collection instruments. Each variable has been given an abbreviation, full name, clearly defined and shown the source of information in terms of where and how to find it. The variable has further been classified as either qualitative or quantitative and either as categorical or discrete or continuous. Each variable has its level of measurement indicated as either nominal, a system for labeling or ordinal, a way of ranking order in relation to each other or interval which means there is a fixed mathematically defined interval. Finally each variable has been given a numerical coding.

4.3.6. *Variables to be collected*

Administrative

- Each questionnaire will have its unique study number
- Each questionnaire will provide dates when interviews were conducted
- Each of the data collectors will have a unique identity code

Demographic

The social demographic variables will include; age, parity, marital status, religious denomination, tribe, place of residence, nearest health facility, level of education, occupation, gender inequalities, barriers to utilization of Antenatal care and health facility based services.

Attitudinal

- Attitude towards health services
- Attitude towards health facility staff
- Cultural, traditional practices and beliefs

Behavioural

- Maternal health seeking behavior
- Perspectives on mother-baby delivery pack
- Place of delivery

Clinical

- Antenatal care utilization during pregnancy
- Delivery preparedness
- Awareness about pregnancy danger signs
- Malaria prevention during pregnancy
- Sources of information about maternal health care services
- Delivery complications

4.4 Operational plan of the study

4.4.1 Activities before field work

1. ***Ethical approval***

   After obtaining ethical clearance from the bio-medical Research and Ethics Committee, final authority to conduct the study will be obtained from the Ministry of Health.

2. ***Oversight***

The principal investigator will be responsible for the management of all aspects of the survey. In view of the large study, the principal investigator will constitute a small team for oversight functions which will include the principal investigator, research assistant, data entry officer and one official each from the central statistics office and District Health Office. The principal investigator will revise and finalise the developed work plan for all the activities in the field and the operational budget.

3. ***Development of work plan***

The work plan will be developed which will provide timetable of activities from the first stages of planning of the survey until after the end of the field work. Key activities in the plan will include;

I. General management
   - Securing and setting up the research office in Monze
   - Recruiting and training of field supervisor, data entry officer and data collectors

II. Logistics management including hiring of vehicles, buying of bicycles and mobile phones, printing of questionnaire, ID cards, checklist for ensuring data completeness and survey materials such as folders, bags for carrying materials for data collectors, pens, erasers, pencils.

III. Questionnaire finalisation
   - Piloting and field testing the questionnaire
   - Final modifications and revision
   - Printing final version of the questionnaire

IV. Sampling

- Setting sample design and frame
- Drawing the sample
- Setting the field work plan
- Listing/Mapping of the primary sampling units (PSUs)
- Assignment of each data collector a portion of the total sample which he/she will be responsible for ensuring that the households in the assigned portion are interviewed

V. Staff Training
- Identification of training venue
- Meeting for trainers to plan
- Arrangements for the training aids (LCD), computer
- Recruiting field supervisor, data entry officer and nine (9) data collectors
- Preparing training manuals
- Training of the research assistant and data collectors

4. *Informing DHMT and Chiefs and the community*

- A copy of the letter from Ministry of Health will be presented to the District Medical Office. Thereafter, with the help of the District Medical Officer, a meeting will be held with the District Medical Office team to introduce and explain the study.

- After holding a meeting with the District Medical office team, a letter will be obtained from the DMO addressed to all the health centre in-charges and all the five (5) Chiefs in the study area, officially informing them about the study.

- A meeting will be held with each chief to further explain the purpose of the study and to request them to inform the respective headmen, who will in turn inform their subjects about the study.

- A meeting with community health workers (CHWs) and neighborhood health committee (NHCs) will be held in each participating health centre to orient them on the study and prepare them to carry out sensitization messages to the community. To ensure that uniform information is given to the community, prepared leaflets with standardized information on the study will be given for distribution in the community.

5. *Recruitment & training of data collectors*

A research assistant, data entry officer and data collectors will be recruited for the study. These will be identified with the help of the Central Statistical Office (CSO).

- The principal investigator will be responsible for the overall conducting of the research, data entry and analysis. The specific tasks will include:

  (i) Logistics arrangements for the study
  (ii) Recruitment of field supervisor, data entry officer and data collectors
  (iii) Organising and conducting of the trainings and piloting of study instruments
  (iv) Organising study launch
  (v) Orientation of the health workers
  (vi) Convening meetings with traditional and local leaders

(vii) Overall responsible for the fieldwork, participation in data collection, supervision, data entry and analysis.

- The research assistant will be responsible for management and supervision of data collection and assist with quality checks of the data. The data entry officer will be responsible for entering data from the questionnaires. Nine (9) data collectors will be recruited from among those who have completed secondary level education and have been trained and involved in surveys in the past by CSO.

- Both the research assistant, data entry officer and the data collectors will receive two-day classroom training by the principal investigator and staff from CSO. The training will include instruction on all study elements amongst them the research proposal, data collection tools, basic health services information. The background information on the study and procedure for collecting information will be given. Every question will be explained in terms of the meaning and how it should be asked. The training will include a session on translating the questions into the local language. The class sessions will also include role-plays and mock interviews. The classroom training will be followed by field training which will involve piloting of the study instruments and a field mock practice in the areas that are not sampled.

- Data collectors will participate in pre-testing of data collection instruments, data analysis and revision of the instruments

- The training and piloting will take one week and the participation of the principal investigator, research assistant and data entry officer in both theory and field training will give them a better understanding of the problems the data collectors may encounter during the field work and also give some experience in working as a team.

6. Conducting a pilot test

The questionnaire will be pre-tested and piloted in the field in the areas that are not sampled. All members of the field team will participate as it will provide training experience, check for problems in the questionnaire design, appropriateness for respondents, clarity, acceptability, lay out and provide an estimate of the amount of time needed to complete the questionnaire. The pre-testing will not only be of the questionnaire but entire fieldwork plan including supervision methods, data entry and written materials such as interviewer manuals, to give assurance in terms of readiness for survey implementation. Members of the field team will meet and discuss the results of the field testing and modify the questionnaire in light of the lessons learned.

7. Launching the study

Two weeks prior to the commencement of the cross-sectional survey, the principal investigator will feature on sensitization programmes on the local community radio station The Principal investigator will give background information about the research, the aim and objectives, importance and what's expected from the community. CHWs and NHCs will also sensitize the community through community meetings at which traditional leaders will be in attendance.

4.4.2 Activities during field work

The actual work of going out to the areas being sampled and data collection in the sampled households will begin one week after the training of field staff. Each data collector will be provided with a mobile phone for communication with the principal investigator and the research assistant, the data collectors will use the mobile phones to

get in touch with the principal investigator and research assistant to seek guidance in case they encounter any problems whilst in the field. Each data collector will also be provided with enumeration area map to help them locate the households and a bicycle for transport in the field. A vehicle will be allocated for both regular and emergency transportation to be used in the field.

1. ***Recruitment of study participants***
   All eligible persons (pregnant women and women who have delivered within the last 10 years in Monze district) found in the selected household will be interviewed.

2. ***Data collection***
   - CHWs, NHCs and local headmen will assist the data collectors in identifying the villages.
   - Using a structured interviewer-administered questionnaire, data collectors will conduct house to house interviews with eligible respondents.
   - Before starting each interview, data collector will introduce himself/herself to the family, and explain the purpose of the study by reading the consent form. After explaining the purpose of the study, the data collector will obtain consent from eligible respondents.
   - In homes where there will be nobody found around, the data collector will leave a note indicating when he/she will return and also leave a message with the neighbours. A maximum of three visits will be made and if they still don't find anybody during the third visit, that household will be put under the non-response group.
   - The households that will decline to be interviewed will also be put under the non response group.
   - Should the data collector encounter any problems in the field, he/she will inform the headman, staff at the health facility, research assistant and the principal investigator.
   - It is anticipated that the survey period will take about one month to be completed.

3. ***Logistical arrangements during field work***

Every morning the data collectors will be taken to their respective enumeration areas and picked up by end of the day. They will proceed to go into the field using bicycles.

4. ***Field Supervision***

The principal investigator and research assistant will be responsible for checking the quality of the work of the data collectors. A checklist will be used by the supervisor in checking completed questionnaire submitted by data collectors and a written record kept. The supervisors will also make un announced visits to areas where data collectors will be operating for the purpose of observing them at work. This will ensure data collectors are where they are supposed to be and also ensure they are following all the procedures taught in the training. Randomly selected households will be re-visited by the supervisors after the eligible member of the household has been interviewed by the data collector.

Any inconsistencies detected while in the field will be resolved by requesting the data collector to return to the household to obtain the correct information. The oversight team will also undertake unannounced visits to the field.

5. Data management during data collection

The data collected and recorded on the forms will be reviewed on daily basis by the field team. This will ensure completeness of forms and making necessary corrections. It will also allow the data collectors to get back the next day for missing fields. The data entry will be done as soon as the questionnaire is completed by the data collectors. The principal investigator and data entry officer will ensure data is entered within the week it’s collected. Validation of data collected will include ensuring all forms submitted for data entry have a date, code and signature of the data collector.

The principal investigator together with members in oversight will meet on weekly basis to review progress, data from the field in terms of completeness and accuracy, address the bottlenecks and challenges in the field.

4.4.3 Activities after fieldwork

The principal investigator will arrange for a debriefing meeting with data collectors immediately after the survey has been completed. Details of the experience, problems encountered, suggestions and recommendations made will be documented. The principal investigator will thank all members of the field team.

4.5 Data Collection and analysis

Pre-tested structured questionnaire will be administered by trained data collectors through face-to- face interviews with pregnant women and women who have delivered within last 10 years in Monze district.

The data will be managed and analysed using statistical Package for Social Sciences (SPSS) packages. A data entry screen will be developed with range and consistence checks embedded in it. Double data entry will be conducted and data files will be validated. The data file in the Epi Info format will be exported to the SPSS format for analysis.

Data will be analysed using logistic regression to determine the effect of various factors on the outcome variables and to control confounding effects. Analysis will look at association of institutional deliveries with age, education, parity, family size, distance from health facility, number of antenatal visits during pregnancy, place of first delivery.

CHAPTER 5

5.0 PROSPECTIVE COMPARATIVE STUDY – (PHASE 2)

5.1. Aim

The aim of the study is to increase institutional deliveries by skilled attendants in the rural areas of Monze District as a key intervention for reducing maternal mortality by addressing hidden costs associated with institutional deliveries through provision of non-financial incentives in the form of mother-baby delivery pack.

5.2. Objectives

To determine the extent to which non-financial incentives in the form of mother-baby delivery pack accompanied with health education and information leaflet contribute to increasing institutional deliveries in rural areas of Monze District over a period of one year, from 1st January to 31st December 2014. Specifically:-

- Determine the impact of non-financial incentive in the form of mother-baby delivery pack on institutional deliveries by comparing intervention and control arms of the study for the period of one year (1st January to 31st December 2014) in rural areas of Monze District.

- Evaluate the effect of non-financial incentives on the number of institutional deliveries by comparing deliveries for the same period in 2012 & 2013 before and 2014 after implementation of the intervention in rural areas of Monze District.

- Document and recommend to Ministry of Health for scaling up if the study shows effect.

5.3. Methodology

5.3.1. *Study site and population*

The study will be carried out in Monze District in Southern Province. The district has a population of 203,038 and 26 health facilities. The district will be stratified into two rural regions separated in the middle by the town centre, one region the western side allocated to intervention arm and the other region the eastern side allocated to the control arm. The study population will be pregnant women in Monze district. The total estimated number of pregnancies in Monze in 2012 was 10,964.

5.3.2. *Study design and study arms*

The study will be an experimental prospective comparative community intervention trial with two comparable rural regions in Monze: The control group will receive routine standard health services provided at health facilities and the intervention arm will receive non-financial incentives in the form of mother-baby delivery pack (napkins, bottle of Vaseline, baby soap, pair of delivery gloves, baby vest, a *chitenge* and an insecticide treated mosquito net) at the time of delivery in a health facility. In addition, pregnant women will have health education sessions during antenatal care visits through the one-to-one and group discussion methods covering various topics. The key messages and information will include the following:-

(i) Birth preparedness

- basic knowledge of pregnancy and infant care
- Identifying health facility for delivery
- Making arrangements for transport for delivery
- Saving money
- Making arrangements in case of emergency

(ii) Danger signs

During pregnancy, childbirth and after delivery

- Illness such as malaria
- Swelling of the feet

- Fitting
- Bleeding from birth canal while pregnant
- Watery discharge before term
- Lack/inadequate foetal movements
- On delivery, presentation with feet or shoulder or hand, cord prolapsed.

Baby

- Difficulty in breathing or not breathing
- Fever, chills, convulsions, rash and jaundice
- Poor sucking or feeding problems
- Vomiting
- Inactive baby
- Red swollen eyes or discharge from the eyes
- Redness, pus or blood from the umbilical cord

Pictorial informational leaflets will be given to pregnant mothers during antenatal care sessions. There will be also community sensitisation using traditional and religious leaders, neighbourhood health centre committees, community health workers and traditional birth attendants. Prior to the sensitisation, an orientation meeting will be held for the mentioned community leaders and health volunteers. The study will cover a period of one year from 1st January to 31st December 2014 in Monze district.

5.3.3. *Power of the study*

Monze District has 35% (2,775) institutional deliveries per year. In order to achieve 80% power to detect a 10% difference in the proportion of non-financial incentive intervention arm vs. control arm at 5% significance level, sample size calculation using WINPEPI gives a total sample required of 752 with 376 in each arm of study. The actual numbers in the study will be 2,775 split into two for each arm of study.

5.3.4. *Outcome measures*

5.3.4.1. Primary Outcome

The primary outcome measure will be the number of institutional deliveries.

5.3.4.2. Secondary Outcomes

(i) Comparison of intervention and control arms

The secondary outcomes will be a comparison of the intervention and control arms on the following:-

a) Birth Preparedness
   - Basic knowledge of pregnancy and infant care
   - Awareness about danger signs during pregnancy
   - Identification of health facility for delivery and transport arrangements to get to health facility for delivery or in case of emergency

b) Maternal complications
   - Haemorrhage
   - Pre eclampsia
   - Eclampsia
   - Obstructed labour
   - Infection
   - Death

c) Baby complications
   - Apgar at birth
   - Gestational age
   - Birth weight
   - Death

d) Mode of delivery and referrals to the hospital

e) Changes in personal determinants i.e. awareness, attitudes.
   - First booking for antenatal care attendance
   - Number of antenatal visits
   - If mother returned for post natal care
   - Timing of post natal care visit
   - If mother took child for children`s under five
   - Timing of children`s under five vi

(ii) Comparison of Pre vs Post Intervention implementation

The secondary outcome measures will also include comparing before and after implementation of the interventions using information routinely collected and recorded in health facility registers. This will include comparing:-

- The age group , young mothers and older mothers
- Primiparous and multiparous
- Presentation at health facility for normal delivery or because of complications (Haemorrhage, pre-eclampsia, eclampsia, obstructed labour, Infection)
- Condition of baby at birth (APGAR score)

5.3.5. ***Sampling Framework***

- The rural health facilities on the western region will be allocated to the intervention arm and the rural health facilities on the eastern region will be allocated to the control arm.

- The two study arms will be separated in the middle by the town centre (District administrative centre) which has the urban health facilities. The town centre will serve as a buffer between the intervention and control arms so that the physical separation of the research arms will prevent the spillover of relevant information on the non-financial incentives package. Crossing the buffer area is very unlikely since it would entail travelling crossing over the buffer area the city centre of more than 30km.

- Each of the health facilities in the intervention arm on the western region will be matched with a health facility in the control arm in the eastern region based on catchment population size and services coverage rates in terms of institutional deliveries.

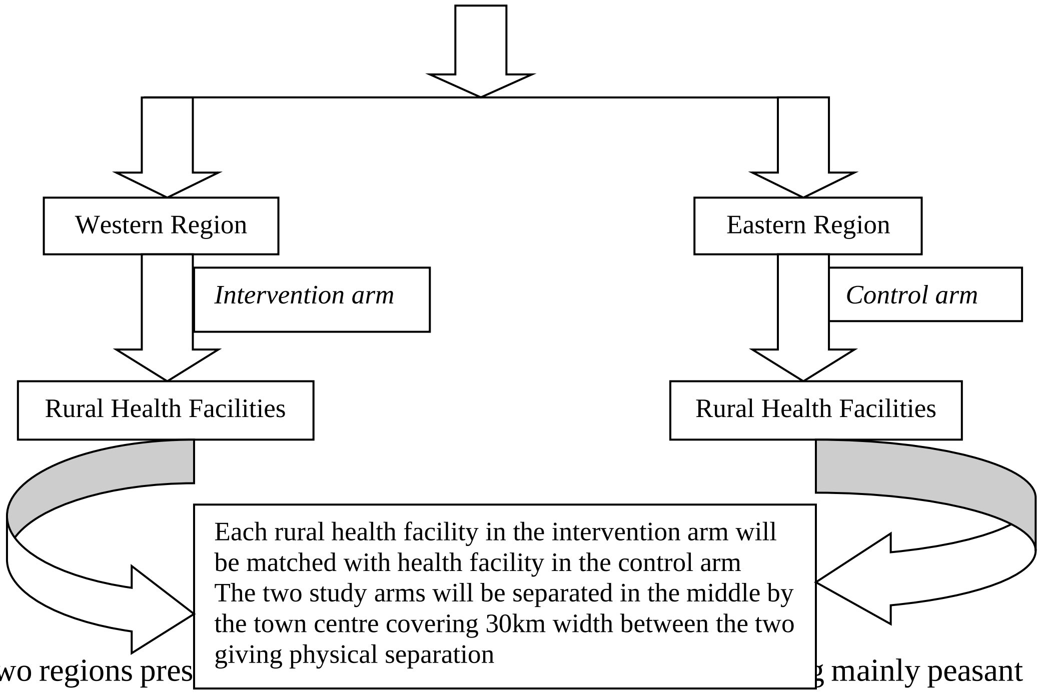

- Both two regions pres[illegible] mainly peasant and subsistence farmers of the same tribe and share same cultural, traditional practices and beliefs and are socio-economically and demographically similar.

- Baseline data on institutional deliveries from 1st January to 31st December for 2012 and 2013 will be collected and recorded from the delivery registers at each health facility in all study sites.

- The standard routine antenatal care health education will be given to all pregnant women both during static and outreach sessions in both arms of study throughout the duration of the study. A sensitisation programme strengthening awareness of the importance of delivering at a health facility by skilled birth attendants, knowledge of danger signs in pregnancy and helping mothers prepare for the delivery are some of the key activities in research sites which will be provided by health centre neighbourhood committees, community health workers, traditional birth attendants, traditional and religious leaders to ensure messages get to all pregnant women.

- In the non-financial incentives intervention arm, the pregnant women will be informed about the mother-baby delivery pack they will benefit from if they deliver at the health facility and information on the content of the pack will be given to the mothers. The mother- baby delivery pack will be given at the health facility when the mother goes to deliver.

- Enrolment will start from 1st January to 31st December 2014.

5.4. Inclusion and exclusion criteria

5.4.1. *Inclusion*

1. Resident in the study area for at least one year
2. Resident in the area until delivery
3. Able to accept the incentive

5.4.2. *Exclusion criteria*

1. Visitor to the region
2. Unable to reside in the area until delivery
3. Unable to accept the incentive

5.4.3. *Study procedures*

1. Administration of the questionnaire
2. Provision of the mother-baby delivery pack as an incentive

5.4.4. *Target population*

Pregnant women in the study sites

5.4.5. *Zones (study sites)*

The study will be carried out in Monze district in Southern Province of Zambia. The district will provide the incentives and services as indicated below but within the district to be stratified in two categories. Each category receiving one set of interventions

1) Provision of non-financial incentives in the form of mother-baby delivery pack (napkins, bottle of petroleum jelly (Vaseline gel), baby soap, pair of delivery gloves, baby vest, a *chitenge* and an insecticide treated mosquito net).

2) Provision of routine standard health services provided in all health facilities in the district (status quo).

5.5. Data Collection and analysis

Data will be collected in two phases, baseline data at the start of the study and prospective data during intervention implementation through two datasheets which will be administered, one for each mother delivering at the health facility in all study sites capturing information on the demographics, the maternal health history, delivery and any delivery complications data. The second health facility information survey questionnaire will capture information from staff and facility records and registers on deliveries, complications, referrals, deaths and on health services provided in general. This will cover the period from 1st January to 31st December 2014. Similar information will be collected for 2012 and 2013 from the registers and records for comparison.

Quantitative data will be managed and analysed using the EPI DATA and SPSS packages, respectively. A data entry screen will be developed with range and consistence checks embedded in it. Double data entry will be conducted and data files will be validated. The data file in the Epi Info format will be exported to the SPSS format for analysis. Proportions will be compared using the Pearson's Chi-square test across the two study arms, while distributions of continuous variables that will be normally distributed will be compared using the Analysis of Variance; otherwise medians will be compared using the Kruskal wallis test.

In assessing the associations of non-financial factors associated with the outcome, the relative risk together with its 95% confidence interval will be used. The cut-off point for statistical significance will be set at the 5% level. The analysis will include comparing the number of institutional deliveries in the intervention and control arms of the study for the period of one year (1st January to 31st December 2014) and comparing the number of institutional deliveries for the same period in 2012 &2013 before and 2014 after implementation of the intervention to determine any statistically significant difference.

5.6. Ethical Consideration

- Approval to conduct the study will be obtained from the Ministry of Health, Zambia.

- Ethical clearance from University College Dublin and the National Health Research Ethics Committee in Zambia will be obtained.

- Individual consent will be obtained from all the pregnant women that will be enrolled in the study, consent forms will be translated in the local language and for those unable to read and write, it will be read to them. The consent form will provide written information on the purpose of the study, procedure, risks, benefits, compensation and issues of confidentiality.

- Participation is voluntary and participants will be free to withdraw from the study at any time.

- In an effort to maintain confidentiality, participants' data will be linked to a code number. Personal data collected will be accessed by the Principal Investigator or with permission from the Principal Investigator.

5.7. Expected Outcomes

- Socio-economic factors associated with institutional deliveries documented, shared with policy makers and stakeholders for evidence-based development of policies, strategies and interventions.
- Effective incentives for institutional deliveries identified.
- Increase in institutional deliveries.
- Reduction in complications associated with child birth as deliveries will be conducted by skilled birth attendants.
- Referral system streamlined and strengthened
- Ultimately contribute to reduction of maternal mortality through addressing some of the barriers to institutional deliveries.

5.8. Use of results if effect found

- Policy formulation and development of in country evidence-based strategies for targeted interventions in rural areas to reduce maternal mortality and morbidity.
- Development of guidelines for service providers.
- Development of a mother-baby delivery pack which could be supplied to rural health facilities on a regular basis similar to Rural Health Centre drug kit
- Scaling-up in a three-phased approach
    - Learning phase to provide evidence (Research sites)
    - Expansion phase (All Provincial centres)
    - Consolidation phase (Countrywide)

5.9. Possible Benefits

- Reduction in maternal mortality
- Reduction in complications associated with non institutional deliveries
- Improved health seeking behavior for maternal health services
- Increase in access to maternal health services by addressing the barriers
- Better outcome for the mother and child
- There will be no direct medical benefits to the participants. However, they will receive a mother-baby delivery pack for those in the non-financial incentives arm to encourage them deliver at the health facility. Others may benefit in future if the information gained in the study leads to reduction in maternal deaths and its related complications.

5.10. Potential Risk

This study will pose no risk to the study participants.

CHAPTER 6

6.0 HUSBANDS' PERCEPTIONS ON PLACE OF DELIVERY IN MONZE DISTRICT, ZAMBIA: A QUALITATIVE STUDY

6.1 Aim

The aim of this qualitative study is to gain understanding of the husbands' role and involvement in the women's decisions regarding the place of delivery in rural Zambia.

6.2 Objectives

1. To explore husbands' perceptions regarding place of delivery in Monze district, Zambia.
2. To determine husbands' role and involvement in the choice of the place of delivery
3. To determine the factors influencing husbands' decision regarding the place of delivery

6.3 Methodology

The qualitative study will be conducted in a natural setting in order to bring out the lived experiences of men regarding women and labour. It will be restricted to the control region so that there is no contamination or spill over from the intervention region who would have received health education and non-financial incentives. The study will describe and interpret daily life experiences and structures (Erlandson, et al., 1993; Creswell, 1998; Denzin & Lincoln, 2000; Neuman, 2004) that shape the lives of both men and women. An ethnographic paradigm will be employed to select the required sampling and data collection methods and weave them into an appropriate methodology.

6.4 Sampling

The approach to the study will be with a worldview of idiocy which is a basic set of beliefs or assumptions that ethnographers or phenomenologists employ in getting into the field. They bracket their preconceptions, experiences, theories and literature. These assumptions help them experience reality at first hand in relation to the nominalist ontology (Blaikie, 2000; Vrasidas, 2000; Hayes and Oppenheim, 1997; Pidgeon and Henwood, 1997; Mills et al., 2006). In this research paradigm, there is the relationship of the researcher to that being researched. The

researcher, in his “humanness,” is part of the research endeavours rather than objective observers, and his values must be acknowledged by readers since this as an inevitable part of the outcome (Appleton, 1997; de Laine, 1997; Guba and Lincoln, 1989; Stratton, 1997). As such, field observations, in-depth interviews and focus group discussions tend to be employed in naturalistic studies (Creswell, 1998: 74) like this one.

Since this study intends to elicit the lived experiences of men relating to maternal health care especially regarding to place of delivery, there is need to enlist men with varying experiences with child birth in order to describe as much as possible what the real life around this phenomenon is. The study will explore the lived experiences of husbands relating to place of delivery, their involvement in the choice of the place of delivery and factors influencing husbands’ decision regarding the place of delivery. This calls for presenting ontologically multiple realities of men i.e., that each *man or husband* has an understanding of reality from an individual perspective (Erlingsson and Brysiewicz, 2013). The study will enlist purposefully men with the following characteristics:

a) Men from a control site, so that there is no contamination or spill over from the intervention region who would have received health education and non-financial incentives.
b) A Chief and a Headman as key informants being the custodian of the norms, history, traditions and cultural practices in the community.
c) Men aged 18 to 34 years (Young men) whose wives (i) delivered at home, (ii) delivered at the health facility.
d) Men aged 35 to 65 years (Adults) whose wives (i) delivered at home, (ii) delivered at the health facility.

These are the typical men who could answer the given research questions. In order to get these men into the study, two types of sampling methods will be used and these are respondent driven sampling and maximum variation sampling.

a) In terms of sampling framework, respondent driven sampling will be employed by enlisting five expectant women from five villages who will come to the health centre for maternal child health services. The number five has been arbitrarily chosen to cover at least five geographical settings to account for variation in experiences. The sampling and recruiting process will begin first with five expectant women acting as seeds with each linking to five other women who have delivered before in Monze district. This cohort of twenty five women will be lobbied to recruit their husbands. It is not anticipated that the first round of RDS will lead to enlisting men with the stated characteristics and to ensure that men with varying experiences or multiple realities are enlisted in the study, men with similar experiences will be excluded to give room for men with a different experience. This will call for the re sampling over and over getting back to the twenty five women with the view to obtain an “ideal” sample (Cresswel, 2007) of men. This shall not be achieved by RDS but by using the second technique which is maximum variation sampling (MVS). With this method we will ensure that sampling will not cease until there is at least men with the following characteristics; a chief, headman, men aged 18 to 34 years (Young men) whose wives (i) delivered at home, (ii) delivered at the health facility and men aged 35 to 65 years (Adults) whose wives (i) delivered at home, (ii) delivered at the health facility.

Through this technique of sampling, it is possible to select respondents and exhibit a wide range of attributes that could elicit unique and common experiences with child birth. The basic principle

behind maximum variation sampling is to gain greater insights into a phenomenon by looking at it from all angles. This means that the participants will be sampled based on particular predetermined criteria or variables in order to cover a range of constituencies (Miles and Huberman, 1994).

6.5 Data collection methods

Data collection in this study will involve use of in-depth interviews.

6.5.1 In-depth Interviews

The principal investigator will make all the necessary arrangements for the in-depth interviews. He will do the necessary clearances at local level, orientation of health facility staff in the study area and recruiting the key informants. He will recruit an assistant fluent in the local language, with experience in conducting qualitative research who will conduct all the interviews. The principal investigator will listen, observe the whole process, record the whole interview and take notes as well. The interview with be semi structured and will begin with a set of baseline questions, but expected to elicit new and perhaps unexpected information by requesting that the key informant expand on his or her answers to these initial questions. This method is ideal for obtaining in-depth descriptive data on beliefs and practices, including historical practices.

6.6 Data analysis

The data analysis will be done by the University of Zambia, Sociology department. Two lecturers in the department, experts in qualitative research will be coders. They will do the data transcription and analysis. The role of the principal investigator will be to ensure inter-coder agreement as well as acquire data analytical skills. Since the data will be in textual form, the data will be reduced and analysed on the computer using N vivo computer package. It should also be noted that computer software for qualitative analysis like N vivo does not analyse data (it is used for organizational support) and the researcher remains the research instrument (Erlingsson and Brysiewicz, 2013:96). Therefore, data reduction process and coding will involve a combination of steps drawn from Hycner (1985:279-303), Stiles and Putnam (1992; 347-355), Rhothstein (1990; 322-327), Boyatzis (1998:4-6) and Gillham (2000:71) as set forth below:

1. **Step 1.** Each recorded interview will be listened to several times in order to have a picture of the general meaning of units or substantive statements. These are words, sentences or paragraphs containing aspects related to each other through their content and context.

2. **Step 2.** All field notes and transcribed interviews will be written and arranged in textual units using the respondent’s literal words. A textual unit is an aggregation of one respondent’s data presented in the form of a complete thought or a sentence.

3. **Step 3.** Each respondent will have a textual unit composed of the interviewer’s questions and the interviewee’s answers. All textual units will be assigned numbers.

4. **Step 4.** Each text will be ascribed a pseudonym for purposes of identification.

5. **Step 5.** Each text will then be read several times by the researcher in order to gain and provide a context for the emergence of demographic characteristics such as: age, education, employment status inter alias and general meaning units.

6. **Step 6**.The text will then be delineated into meaning units by going over every word, phrase, sentence and paragraph. This is the process of getting at the essence of the

meaning expressed in a word or phrase or sentence or paragraph. The meaning unit sheds light on a specific explicit area of content, which is identified with little interpretation. A meaning unit can be parts of the text based on theoretical assumptions from the literature, or parts of the text that address a specific research question or topic in an interview or observation. A meaning unit is the crystallization or condensation of what the social actors would have said. This is because a unit of meaning expresses a unique and coherent meaning. It is from this process that coding of themes; headers and sub headers can be created. According to (Coffey and Atkinson, 1996) `codes are tools to think with' and `heuristic devices' since labeling a condensed meaning unit with a code allows the data to be thought about in new and different ways. A code can be assigned to, for example, discrete objects, events and other phenomena, and should be understood in relation to the context.

7. **Step 7.**Themes will be selected inductively from raw information on the basis of observed phenomena or deductively generated. Phenomena will have information at a minimum to describe and organise the possible observations and at a maximum, to interpret aspects of the phenomenon.

8. **Step 8.** Headers will then be selected to fit the themes. Creating themes is a way to link the underlying meanings together in categories. A theme is a recurring regularity developed within categories or that cuts across categories.

9. **Step 9.** Sub headers will then be defined considering that headers will have more than one descriptive organised element.

10. **Step 10.** Essentially sub headers will be linked to the questions asked.

11. **Step 11**. From the sub headers, themes, which have a relevance to answering research questions, will be identified and be delineated and deposited into appropriate nodes. Nodes are the main categories of research issues. Categories are the core feature of qualitative content analysis. A category is a group of content that shares a commonality. Boyatzis (1998) and Gillham (2000) emphasise that categories must be exhaustive and mutually exclusive. This means that no data related to the purpose should be excluded due to lack of a suitable category. Furthermore, no data should fall between two categories or fit into more than one category.

12. **Step 12.** Two lecturers from the University of Zambia, Sociology department, experts in qualitative research will be the coders. In order to ascertain inter-coder reliability between each of the two coders during the theme location and the manual coding stage, the coders will work in a rotation (round robin) whereby each would have an opportunity to write themes and code all responses and observations. The principal investigator will be available throughout the themes location and the categorical coding duration to provide for reduction reliability spot checks. This will cover comparing all categorized themes between the two coders. This process will create an incentive for attention to the task of coding and instantaneous checking.

Having done so, then the analysis could by induction and deduction continue along the nodal trees and by doing this, it is easy to show any phenomena of interest, to create typologies which will aid in presenting answers to the research questions. From this process, it would be possible to uncover the invariant features of phenomena in order to provide a rigorous description of them by looking for common themes and to write a composite summary of what the scenario and perceptions of men are like in terms of roles and involvement of men in improving institutional deliveries in the rural parts of Monze is like.

6.7 Activities before field work

6.7.1 Ethical Consideration

- Approval to conduct the study will be obtained from the Ministry of Health, Zambia.

- Ethical approval will be obtained from the National Health Research Ethics Committee in Zambia. After obtaining approval from Zambia, an application for exemption from further review will be made to the UCD Ethics Committee

- Individual consent will be obtained from all men that will be enrolled in the study, consent forms will be translated in the local language and for those unable to read and write, it will be read to them. The consent form will provide written information on the purpose of the study, procedure, risks, benefits, compensation and issues of confidentiality.

- Participation is voluntary and participants will be free to withdraw from the study at any time.

- In an effort to maintain confidentiality, participants' data will be linked to a code number. Personal data collected will be accessed by the Principal Investigator or with permission from the Principal Investigator.

6.7.2 Study period

The study will take place in October to December, 2014.

Operational Plan and Timelines

June to August
- Develop the qualitative research protocol

- Clear with the Supervisor and DSP

- Link up with the local university in Zambia, Sociology department for possible collaboration in conducting the qualitative study

August/September
- Submit to the National Health Research Ethical Committee for clearance and approval

- Apply to the UCD Ethics Committee for exemption

- Procure items (Recorders, tapes and stationery)

October/December
- Recruitment of assistant who will conduct interviews

- Conduct sensitization and community meetings in study areas
- Pilot the topic guide
- Conduct in depth and Key Informant Interviews

January/May 2015

- Transcription
- Conduct analysis of the descriptive data

Bibliography

Appleton, J. (1997). Constructivism: A naturalistic methodology for nursing inquiry. Advances in Nursing Science, 20(2), 13-22.

Blaikie, N. (2000). Designing Social Research: The Logic of Anticipation. Malden: Blackwell Publishing Inc.

Boyatzis, R.E. (1998). Transforming Qualitative Information. Thousand Oaks. Sage Publications.

Coffey, A., and Atkinson,P. (1996). Making Sense of Qualitative Data. Complementary Research Strategies. Thousand Oaks. Sage Publications Inc.,

Creswell JW. (2007) Qualitative Inquiry and Research Design: Choosing Among Five Traditions. London: Sage Publications.

Creswell, J. (1998). Qualitative inquiry and research design: Choosing among five traditions. Thousand Oaks,CA: Sage.

de Laine, M. (1997). Ethnography: Theory and applications in health research. Sydney, Australia: Maclennan and Petty.

Denzin, N. K., & Lincoln, Y., S. (2000). Introduction: The discipline and practice of qualitative research. In N. K.

Erlandson, D. A., Harris, E. L., Skipper, B. L., & Allen, S. D. (1993). Doing naturalistic inquiry: A guide to methods. Newbury Park, CA: Sage.

Erlingsson, C., and Brysiewicz, P. (2013). Orientation among multiple truths: An introduction to qualitative research. African Journal of Emergency Medicine. 3: 92–99.

Gile, K. J. (2011). Improved Inference for Respondent-Driven Sampling Data With Application to HIV Prevalence Estimation. Journal of the American Statistical Association 106 135-146.

Gillham, B. (2000). Case Study Research Methods. London. Continuum.

Guba, E., and Lincoln,Y. (1994). Competing paradigms in qualitative research. In N. Denzin and Y. Lincoln (Eds.), Handbook of qualitative research (pp. 105-177). Thousand Oaks, CA: Sage.

Hayes, R., and Oppenheim, R. (1997). Constructivism: Reality is what you make it. In T. Sexton and B. Griffin (Eds.), Constructivist thinking in counselling practice, research and training (pp. 19-41). New York: Teachers College Press.

Heckathorn, D. D. (2002). Respondent-driven sampling II: Deriving valid population estimates from chain-referral samples of hidden populations. Social Problems 49 11-34.

Hycner, R.H. (1985). Some Guidelines for the Phenomenological Analysis of Interview Data. Human Studies**. 8**: pp.279-303.

Miles M, Huberman M. (1994) Qualitative Data Analysis: An expanded Sourcebook. Beverly Hills, CA: Sage Publications.

Mills, J., Bonner, A., Francis, K.(2006) The Development of Constructivist Grounded Theory. International Journal of Qualitative Methods. 5(1): 25-35.

Neuman, W. L. (2004). Basics of social research: Qualitative and quantitative approaches. Boston: Pearson Education.

Pidgeon, N., and Henwood, K. (1997). Using grounded theory in psychological research. In N. Hayes (Ed.), Doing qualitative analysis in psychology (pp. 245-273). Hove, UK: Psychology Press.

Rhothstein, R.H. (1990). Inter rater reliability of job performance ratings: Growth to asymptotic level with increasing opposition to observe. Applied Psychology. **75**: pp.322-327.

Schonlau M, Liebau E. (2012) Respondent-driven sampling, The Stata Journal. 12(1): 72-93.

Stiles, W.B., and Putnam, S. (1992). Verbal exchanges in medical interactions: Concepts and measurement. Social Science and Medicine. **35**: pp. 347-355.

Stratton, P. (1997). Attributional coding of interview data: Meeting the needs of long-haul passengers. In N. Hayes. (Ed.), Doing qualitative analysis in psychology (pp. 115-141). Hove, UK: Psychology Press.

Van Manen M. (1990) Researching lived Experience: Human Science for an Action Sensitivity Pedagogy. London: Althouse Press.

Vrasidas, C. (2000). Constructivism versus objectivism: Implications for interaction, course design, and evaluation in distance education. International Journal of Educational Telecommunications, 6(4), 339-362.

CHAPTER 7

7.0 DETERMINATION OF TOTAL DELIVERIES IN ONE RURAL HEALTH CENTRE CATCHMENT POPULATION

7.1. Birth Notification in Monze District

Zambia and Monze district in particular does not have a legal requirement or system for collection of information on home deliveries. As such, there is no routine data on births and deaths currently being collected from the community. The only available information on births is collected through health facilities and that is what is currently recorded as institutional deliveries. The country has not yet made it mandatory for all the births and deaths to be notified. However, discussions have commenced at policy level to put in place a system for collection of information on home deliveries at community level. Currently only institutional data on deliveries is available in Monze. There is a lot of secrecy regarding births and deaths in rural areas especially on miscarriages and newborns. In most instances information is concealed due to traditional beliefs and myths. Therefore, there are challenges in getting data on home deliveries in all the study sites. The challenges include:-

- No system or structures for collection of data at community level.
- Headmen lack capacity to identify households with home deliveries as there is no streamlined reporting system from the village to the headman and ability to record this information as most of the headmen are not educated (unable to read or write), they may have to depend on someone possibly a school-going child within the household to write for them.
- No source of funding for undertaking collection of information on home deliveries. The exercise requires adequate resources to ensure completeness of the information, provision of incentives, transport for the elderly women or family members that assist pregnant women to deliver in homes to take information to the headmen and health facility.
- Physical barriers such as mountains and rivers in some parts of Monze which make it a very difficult terrain with poor road network and scattered population compounded with long distances.

The United Nations Inter-agency group (WHO, UNICEF, UNFPA, World Bank) has developed guidelines for developing countries to use for calculating expected pregnancies and estimating deliveries. This was as a result of challenges countries were having in collecting

home delivery information. Zambia has adopted the guidelines and uses the following formulas:-

- Expected pregnancies 5.4% of the population
- Expected deliveries 5.2% of the population.

In this study I have no capacity to cover all the study areas to collect information on home deliveries due to scattered population compounded with long distances since Monze is farming community, people are sparsely populated. There are no enough resources to meet the financial requirements to do the exercise.

7.2. Home delivery data for the study

Part of the study includes comparing and validating the formula currently being used. In order to collect data on home deliveries, a small study restricted to only one rural health centre catchment population will be conducted. The data on both home and institutional deliveries will be collected for Keemba Rural Health Centre. The health centre is located in the intervention arm and has the following demographics for 2013:-

- Catchment population of 13 580
- Expected deliveries 706
- 1st ANC attendance was 451 (62%)
- Institutional deliveries were 233 (33%)

The home and institutional delivery data for Keemba Rural Health Centre catchment population will allow comparison and validation of the formula by the United Nations Inter-agency group currently being used in Zambia, which includes study sites to estimate total deliveries. Data collection on total deliveries for 2014, both home and institutional in Keemba Rural Health Centre catchment population has been planned for June to August 2015.

APPENDICES

Appendix A

PROGRAMME OF WORK & TIMELINES

Improving Institutional Deliveries in Rural Zambia

Research Proposal for PhD in Public Health

Dr. Victor M Mukonka

Submitted to
University College Dublin
2013

YEAR ONE (2013) – PREPARATORY PHASE

February (Ireland)

- Arrival in Ireland
- Registration & Administrative
- Report to the school & Orientation, Christine Ridge
- Meeting the Director PhD Programme, Dr Mary B. Codd
- Working on the Research Proposal and instruments for data collection

March (Ireland)

- Meeting the Director PhD Programme, Dr Mary B. Codd
- Meeting the Supervisor, Dr Patricia Fitzpatrick and discuss the research proposal.
- Attend PHPS 40620 (Graduate Seminar)
- Working on the Research Proposal and instruments for data collection.

April (Ireland)

- Weekly meeting with the Supervisor (Dr Patricia Fitzpatrick)
- Working on the Research Proposal and instruments for data collection
- Attend PHPS 40620 (Graduate Seminar)

May (Ireland)

- Working on research proposal.
- Working on instruments to be used in the field
- Meeting the Doctoral Studies Panel
- Present research proposal to Graduate Seminar
- Submit application to UCD for research ethical approval consideration for June sitting.
- Attend PHPS 40620 (Graduate Seminar)
- Attend & Speak at Women Deliver & Countdown to 2015 MDGs Conference in Kuala Lumpur

June – August (Zambia)

- Submission of the Research Protocol to the Research and Ethics Committee (REC) for Ethical Approval in Zambia
- Ministry of Health approval of the research study

- Engage the Ministries of Health and Community Development, Mother and Child on the study
- Tendering and procurement of the mother-baby delivery packs.
- Present research proposal to the Copperbelt University School of Medicine
- Make presentation at Tropical Diseases Research Centre (TDRC) scientific seminar.
- Engage and meeting with WHO, UNICEF, UNFPA & World Bank on the study
- Discussions and collaborations with the local USAID research institutions
- Hold inception meeting with Monze District Medical Office
- Continue with the writing up and literature review
- Recruitment and training of Research Assistants

- Orientation of health facility staff, TBAs and other community-based individuals on the study and information to be given to women during health talks.
- Piloting study instruments and feasibility studies

September (Ireland)
- Finalize preparatory phase ie proposal & tools
- Attend Applied Research Methods & Data Management module PHPS 40550
- Attend PHPS 40620 (Graduate Seminar)

October – 11th November (Ireland)
- Meeting with Doctoral Studies Panel
- Attend PHPS 40550 (Applied Research Methods & Data Management)
- Attend PHPS 40620 (Graduate Seminar)
- Final Assessment for PHPS40550 (Class presentation)

12th November – December (Zambia)
- Sensitization program in research sites
- Setting up and preparing research sites
- Finalise preparations for the data collection and conduct the pilot test in the field.
- Set up and conduct the cross-sectional survey in November and December 2013.
- Do preliminary analysis of the data.

YEAR TWO (2014) - IMPLEMENTATION PHASE

- Setting and conducting the prospective comparative community intervention trial from 1st January to 31st December 2014 in Monze District, Southern Province of Zambia.
    - Collect baseline data on institutional deliveries from 1st January to 31st December for 2012 and 2013.
    - Provide health education during antenatal static and outreach sessions in the intervention arm of the study throughout the duration of the study.
    - Sensitisation program in research sites by Neighbourhood health committees, Community Health Workers, Traditional Birth Attendants, traditional and religious leaders throughout the duration of the study.

- The intervention arm receives a mother-baby delivery pack as non-financial incentive. The control arm receives routine care. The pack will be opened at the time of delivery.
- Administration of structured questionnaire to pregnant women delivering at health facility capturing information on the demographics, maternal health history, delivery and any delivery complications data.
- Administration of second health facility information survey questionnaire capturing information from staff and facility records and registers on deliveries, complications, referrals, deaths.
- Collected data entered and cleaned
- Preliminary analysis of the data.

May - June (Ireland)

- Meetings with Supervisor
- Attend PHPS 40620 (Graduate Seminar)
- 3rd DSP meeting Friday, 9th May 2014 at 11hrs before appearing for stage transfer assessment.
- Make pre-transfer assessment presentation during the graduate seminar on 15th May 2014.
- Attend Faculty of Public Health Medicine Summer Scientific Meeting, 21 – 22rd May 2014 and make poster presentation.
- Appear for stage transfer assessment on Wednesday 11th June 2014 at 11hrs.
- Attend U21 European MDG workshop on 13 – 14th June 2014 and make a presentation on Friday 13th June 2014 at 15hrs.
- Leave for Zambia on 20th June 2014.

July - December (Zambia)

- One trip by supervisor to research sites in Monze District, Zambia
- Conduct Qualitative study on male perceptions and attitudes to institutional and home deliveries in Monze (Nov/Dec, 2014).
- Complete implementation of the one year prospective community intervention trial data collection (January 2014 to December 2014).
- Carry out detailed analysis of the cross-sectional data to establish statistically significant barriers and factors to institutional deliveries.
- Continue working on literature review.
- Arrange the 4th DSP Meeting.

YEAR THREE (2015) - FINALISATION PHASE

January – May (Ireland)

- Meetings with Supervisor
- Arrange 5th Doctoral Studies Panel Meeting
- Take Advanced qualitative Design and Analysis Module PSY40220 from January to April 2015

- Take Statistical Modelling and Survival Analysis Module PHPS40460 from January to April 2015
- Undertake NVIVO training (Jan – April, 2015).
- .Attend Graduate Seminar (PHPS 40620) every time I am in Ireland, specifically the Jan, Feb, March, April, May Graduate Seminars.
- Data analysis, interpretation and thesis writing
- Writing Manuscripts for Publications

June – August (Zambia)

- Conduct mini census to collect data on both home and institutional deliveries for 2014 in at least one rural health centre. This data will be used to compare and validate the formula by the United Nations Inter-agency group currently being used in Zambia to estimate total deliveries.
- Data analysis, interpretation and thesis writing
- Presentation to research participants
- Presentation to stakeholders in Zambia
- Writing Manuscripts for Publications

September – December (Ireland)

- Meetings with Supervisor
- Arrange 6th Doctoral Studies Panel Meeting
- Attend Graduate Teaching Assessment Module – Tutor for Final Med PHPS30020
- Attend PHPS40620 (Graduate Seminar)
- Finalise data analysis, interpretation and thesis writing
- Mock presentation to the Graduate Seminar
- Final submission in December 2015
- Defense and Dissemination

REFERENCES

Alliance Zambia. March 2012 http://www.aidsalliance.org/NewsDetails.aspx?Id=291234

Alvarez, J., R. Gil, V. Hernández, and A. Gil, 2009, Factors associated with maternal mortality in Sub-Saharan Africa: an ecological study: BMC Public Health, v. 9, p. 462.

Bhutta, Z. A., M. Chopra, H. Axelson, P. Berman, T. Boerma, J. Bryce, F. Bustreo, E. Cavagnero, G. Cometto, and B. Daelmans, 2010, Countdown to 2015 decade report (2000–10): taking stock of maternal, newborn, and child survival: The Lancet, v. 375, p. 2032-2044.

Buor, D., and K. Bream, 2004, An analysis of the determinants of maternal mortality in sub-Saharan Africa: Journal of Women's Health, v. 13, p. 926-938.

CSO, 2010, Census of Population and Housing Report 2010, Lusaka, Zambia, Central Statistical Office.

Ekirapa-Kiracho, E., P. Waiswa, M. H. Rahman, F. Makumbi, N. Kiwanuka, O. Okui, E. Rutebemberwa, J. Bua, A. Mutebi, and G. Nalwadda, 2011, Increasing access to institutional deliveries using demand and supply side incentives: early results from a quasi-experimental study: BMC international health and human rights, v. 11, p. S11.

Gabrysch, S., and O. M. R. Campbell, 2009, Still too far to walk: literature review of the determinants of delivery service use: BMC Pregnancy and Childbirth, v. 9, p. 34.

Gabrysch, S., S. Cousens, J. Cox, and O. M. R. Campbell, 2011, The influence of distance and level of care on delivery place in rural Zambia: a study of linked national data in a geographic information system: PLoS medicine, v. 8, p. e1000394.

Gage, A. J., 2007, Barriers to the utilization of maternal health care in rural Mali: Social Science & Medicine, v. 65, p. 1666-1682.

Gopalan, S., and V. Durairaj, 2012, Addressing maternal healthcare through demand side financial incentives: experience of Janani Suraksha Yojana program in India: BMC Health Services Research, v. 12, p. 319.

HMIS, 2012, Health Management Information System, Monze, Zambia, Ministry of Health.

Ir, P., D. Horemans, N. Souk, and W. Van Damme, 2010, Using targeted vouchers and health equity funds to improve access to skilled birth attendants for poor women: a case study in three rural health districts in Cambodia: BMC Pregnancy and Childbirth, v. 10, p. 1.

MNCH, 2012, Maternal, Newborn & Child Health Road Map for Zambia, Lusaka, Zambia, Ministry of Health.

NHP, 2012, National Health Policy - August 2012, Lusaka, Zambia, Ministry of Health, Zambia.

NHSP, 2011, National Health Strategic Plan 2011 - 2015, Lusaka, Zambia, Ministry of Health.

Palamuleni, M., 2011, Determinants of non-institutional deliveries in Malawi: Malawi Medical Journal, v. 23, p. 104-108.

RBF, 2011, Operational Implementation Manual for Results Based Financing (RBF) in Pilot Districts in Zambia - 14th November 2011, Lusaka, Zambia.

Ronsmans, C., and W. J. Graham, 2006, Maternal mortality: who, when, where, and why: The Lancet, v. 368, p. 1189-1200.

Shiferaw, S., M. Spigt, M. Godefrooij, Y. Melkamu, and M. Tekie, 2013, Why do women prefer home births in Ethiopia?: BMC pregnancy and childbirth, v. 13, p. 5.

Soyoola, M., and E. Banda, 2010, Report of a Rapid Demand Side Assessment of Barriers to Use of Maternal and Newborn Services and Care in Choma District, Southern Province, Zambia - August 2010, Lusaka, Zambia.

Soyoola, M., and N. Ruth, 2011, Report of a Rapid Demand Side Assessment of Barriers to Use of Maternal and Newborn Services and Care in Chama District, Eastern Province, Zambia - June 2011, Lusaka, Zambia.

Stekelenburg, J., S. Kyanamina, M. Mukelabai, I. Wolffers, and J. Roosmalen, 2004, Waiting too long: low use of maternal health services in Kalabo, Zambia: Tropical Medicine & International Health, v. 9, p. 390-398.

Surridge, M., and L. Mate, 2011, Report of a Rapid Demand Side Assessment of Barriers to Use of Maternal and Newborn Services and Care in Kaoma District , Western Province, Zambia - May 2011, Lusaka, Zambia.

Thaddeus, S., and D. Maine, 1994, Too far to walk: maternal mortality in context: Social science & medicine, v. 38, p. 1091-1110.

Varma, D. S., M. E. Khan, and A. Hazra, 2010, Increasing institutional delivery and access to emergency obstetric care services in rural Uttar Pradesh: J Fam Welf, v. 56, p. 23-30.

ZDHS, 2007, Zambia Demographic and Health Survey 2007 Report, Lusaka, Zambia, Central Statistical Office.
