# Supplementary material for: Effect of a mother-baby delivery pack on institutional deliveries: A community intervention trial to address maternal mortality in rural Zambia
Source: PLoS One. 2024 Mar 11;19(3):e0296001. doi: 10.1371/journal.pone.0296001 (PMC10927137; doi:10.1371/journal.pone.0296001)
Supplement: S2 File — (PDF) [file pone.0296001.s004.pdf]

TROPICAL DISEASES RESEARCH CENTRE
Tel/Fax +260212615444
tdrc-ethics@tdrc.org.zm
P O Box 71769
NDOLA, ZAMBIA

TDRC ETHICS REVIEW COMMITTEE
IRB REGISTRATION NUMBER: 00002911
FWA NUMBER: 00003729

TRC/ERC/04/09/2013

10th September, 2013

Dr. Victor Mukonka
CBU School of Medicine
P.o Box 71191

Ndola

Dear Dr. Mukonka,

Sub: Improving Institutional Deliveries in Rural Zambia

Reference is made to the protocol entitled **"Improving Institutional Deliveries in Rural Zambia."**

On behalf of the Chairman of the TDRC Ethics Review Committee, I wish to inform you that your research protocol was reviewed and your responses to the comments that were earlier raised by reviewers were thoroughly attended to.

I, therefore, inform you that your protocol has been approved.

We wish you every success in the execution of this important study.

Yours Sincerely
TROPICAL DISEASES RESEARCH CENTRE

TROPICAL DISEASE RESEARCH CENTRE
ETHICS REVIEW COMMITTEE
10 SEP 2013
P.O.BOX 71769
NDOLA, ZAMBIA

Eric M. Njunju Bsc, Msc
SECRETARY, TDRC ETHICS REVIEW COMMITTEE
