# Supplementary material for: Effect of a mother-baby delivery pack on institutional deliveries: A community intervention trial to address maternal mortality in rural Zambia
Source: PLoS One. 2024 Mar 11;19(3):e0296001. doi: 10.1371/journal.pone.0296001 (PMC10927137; doi:10.1371/journal.pone.0296001)
Supplement: Attachment — Submitted filename: Responses to Reviewers.pdf [file pone.0296001.s007.pdf]

Responses to Reviewers' comments

Reviewer # 1: Minor revision:

Query 1: Line 61: R-studio is a development environment for R. It is important to state the version of R that was used for conducting the analysis. In fact, the use of R-studio is rarely noted in manuscripts.

Response: We appreciate the comment from the reviewer. We mentioned in the abstract and methods sections that the Analysis was conducted using R-studio statistical software version 4.2.1 (**see pages and 7**).

Query 2: Line 59: Chi-square and independent t-tests are used to make comparisons between arms. Be sure to include the word "compare" or "comparisons" in this sentence.

Response: We appreciate the observation; we have made the change accordingly (**page 2)**

Query 3: Line 61: Clarify if "(p-value < 0.05)" implies that p-values < 0.05 were considered statistically significant.

Response: We appreciate the comment. We have now clarified that the p-value<0.05 was considered significant (**see page 2 and 7**).

Query 4: Line 198: The independent t-test and chi-square tests are actually inferential tests rather than descriptive statistics. Descriptive statistics are means, standard deviations, frequencies, and percentages, etc.

Response: We thank the reviewer for the comment. We have now corrected the sentence to read: "Descriptive statistics (frequencies, percentages, proportions, means and standard deviations) was used to summarise participant socio-demographic and clinical data as well as institutional deliveries in intervention and control areas for each year" (**see page 7**)

Query 5: Supplementary Table 1 contains replicates of the variables.

Response: We have corrected the table and removed all the replicate variables (**see table 1**).

Query 6: Supplementary Tables 2 and 3: State a more precise p-values rather than > 0.05.

Response: The tables have been corrected accordingly (**see tables 2 and 3)**

Reviewer #2: General:

Query 1: The introduction is too brief and lacks key information for the reader. There is no information on ANC or on related interventions and their effectiveness. What have we already learned that led the authors to design and test this specific intervention? There should be sufficient information that a reader can glean this from the introduction.

Response: We thank the reviewer for the insightful feedback. We have edited the introduction and added a section on antenatal care [**see pages 4 and 5)**

Query 2: This manuscript reports findings from a randomized trial. The CONSORT guidelines should be used to ensure all relevant information is included. I suggest the authors download the CONSORT checklist and verify all information is included.

Response: We appreciate the reviewer's comment. The CONSORT guidelines was used and filled in accordingly

Query 3: The statistical analysis is not appropriate for the trial design. This was a cluster randomized trial; therefore, facility-level clustering needs to be accounted for in the analysis or standard errors will not be approrpriately estimated, potentially biasing the results and leading to incorrect interpretation. I have provided more details below. Moreover, characteristics that were different between arms at baseline should be adjusted for.

Response: We thank the reviewer for the comments. The respondent characteristics at baseline were compared between the intervention and control sites (see table 1). There was no significant difference between the two groups with regard to age, number of children and antenatal care booking. A significant difference between the two groups with regard to gravidity. Stratified analysis of variance ANOVA comparing the mean number of deliveries before and after the intervention in the same facilities accounts for any potential facility-level clustering. Moreover, we have explained that both intervention and control regions had similar health facilities regarding the location (rural), size and catchment population, socio-economic and demographic profiles. The two regions mainly served peasant and subsistence farmers of the same tribe who shared same cultural, traditional practices and beliefs. To ensure that the two regions were comparable with regard to population size, population data for the two regions was obtained from the Central Statistical Office in Zambia (**see page 6).**

Query 4: There should be some analysis of participants lost to follow up. Are these women significantly different? How might this bias results? I cannot fully evaluate the results or discussion at this time given the limitations of the methods (lack of information).

Response: We appreciate the comment from the reviewer. We mentioned that a total of 5,000 pregnant women were initially recruited into the study; meaning that they expressed interest and willingness to participate in the study. This was done at first contact in the antenatal care clinic. However, for various reasons, not clearly understood, 500 (10%) never returned to the health facility for follow up. Thus, they were deemed not part of the study since they were not exposed to the study and information on these is not available (**see page 10**).

Query 5: Were women not exposed to the intervention at different points of their pregnancy? The authors should expand on the timing of outcome measures relative to the intervention. If women were enrolled at their first ANC visit, it is unclear to me whether they were observed after the conclusion of the intervention as this is not clear from the methods section.

Response: We thank the reviewer for the insightful comment. Yes, women were identified from the ANC clinics, when they went for their first ANC visit, regardless of the stage of their pregnancy (gestation). The outcome of interest was coming to the health facility for delivery (institutional delivery). Thus, their follow up ended when they delivered; either at the health facility or home. They were not observed after the intervention. To ensure that no woman gave birth after the end of the follow up period, care was taken during recruitment to ensure that only women whose expected date of delivery (EDD) fell before 30th Nover, 2014 were recruited into the study. We picked on 30th November to allow for one month window for women who would experience prolonged gestation beyond 40 weeks to be observed. Participant recruitment ended in mid- year (June, 2014) so that the last delivery would be expected before the end of the intervention in December, 2013. In this case, no further follow up was not needed after the study. Moreover, this avoided the need for right censoring in our analysis (**see page 8**).

Abstract:

Query 6: Please define secondary outcomes reported as results here.

Response: We have made the correction accordingly (**see page 2**)

Query 7: The abstract attributes the difference in arms to the mother-baby pack, but the abstract notes that the intervention also included health education. Is the difference attributed to both or can the authors statistically isolate the effect of the mother-baby pack? Either way, this should be clear in the abstract

Response: We have corrected the conclusion section in the abstract to include. It now reads as follows: "*These findings provide evidence for the effectiveness of the mother-baby delivery pack and additional health education sessions on increasing institutional deliveries in rural Zambia*" (**see page 2**).

Introduction:

Query 8 : Line 82-83: "Home deliveries…" this sentence makes a claim that should be supported by a citation(s).

Response: We have included the citations accordingly (**see page 4**)

Query 9: Why is there no information or literature review on ANC in the introduction if this is a key component of the intervention evaluated? Please describe relevant background on ANC, including number of recommended visits and attendance rates (e.g., from the most recent DHS) in Zambia, and barriers to ANC (could be more broadly, as relevant to the study population).

Response: We thank the reviewer for the thoughtful comment. We have added information on ANC in Zambia (**see page 4 and 5**)

Methods:

Query 10: Are there any private facilities that can support deliveries?

Response: We have edited the section on the study setting and added more detail on the health facilities in the district. We have also mentioned that, at the time, there were no private health facilities providing obstetric and newborn care in the district (**see page 6**).

Query 11: The secondary outcome measures need to be more clearly defined. For example, "knowledge of pregnancy danger signs" is vague.

Response: We have edited the sentence to read: "The secondary outcome measures were: 1) antenatal care service utilisation; 2) postnatal care service utilisation by mother and baby; 3) under-five clinic service utilisation

Query 12 : How was the randomization conducted?

Response: We used cluster sampling; allocation was done at the cluster/ rural region level; the district was stratified into two rural regions separated in the middle by the town centre. The region on the western side was allocated to the intervention arm; the one on the eastern side as the control arm. Eight health facilities were included in each arm (**see page 6 under trial design and randomisation**)

Query 13: Information about recruitment should be in the enrolment section (around line 167).

Response: This has been noted and corrected accordingly (**see page 7)**

Query 14: The abstract states analysis was done in R but the methods section states analysis was conducted in SPSS. Please clarify so that this is consistent.

Response: This was an error. We have now corrected it to read, "analysis was conducted using R-studio statistical software version 4.2.1" (**see page 9**).

Query 15: The information about data collection is insufficient. Please state where 2012 and 2013 data came from, and exactly what data was collected (e.g., precisely what measures were on the data extraction sheet, who extracted data).

Response: We thank the reviewer for the insightful question and comment. We have now explained that both the baseline and intervention data were collected by a pair of data collectors under the supervision of the principal investigator. In order to establish baseline delivery data prior to commencement of the intervention, year-long delivery records for 2012 and 2013 were reviewed and delivery data collected from the delivery registers at each health facility in the study sites, using a data extraction sheet. The data extraction sheet comprised various sections including demographics (age, place of residence), gravidity, parity, gestation, pre-existing medical conditions (hypertension, HIV, anaemia), expected date of delivery, date of delivery, place of delivery, mode of delivery, delivery outcome, baby condition and outcome (**see page 9**).

Query 16: What is the timing of paper-based questionnaires ("PNC" is far too vague, for example).

Response: We have explained that the paper-based questionnaire data was collected at 3 time points: at enrolment into the study during the ANC clinic, in the labour ward when the woman came for delivery and when the woman and baby came for the first PNC visit (**see page 9**)

Query 17: How were interviewers trained? Where were interviews conducted?

Response: We thank the reviewer for the comment. We have provided a detailed explanation on how the data collectors were trained: the training lasted 4 days (3 days three and 1 day practical). No interviews were conducted; rather a structured questionnaire was administered by midwives who were trained as data collectors. Above we have explained when and where the data was collected from (in the ANC, delivery and PNC clinics (**see page 9**)

Query 18: All non-descriptive analyses require clustering of the standard errors by facility given the study design. The authors should use regression models (e.g., linear, logistic depending on the outcome) with standard errors clustered by health facility. Otherwise, the independence assumption is violated which will bias standard errors and thus potentially lead to incorrect inferences about the statistical significance of the estimated relationships. Regression models estimating the effect of the intervention should also adjust for factors that were significantly differnet between arms at baseline, such as gravidity. Otherwise, this may be a spurious relationship.

Response: We thank the reviewer for the observation and comment. The aim of the tudy was to determine the effect of provision of additional health education during antenatal care (ANC) and a non-financial incentive on institutional deliveries. In our view, this effect can be tested using different models: analysis of various (ANOVA) or regression (linear, logistic or Poisson). In this case, we opted for one way ANOVA. We compared the mean number of deliveries between the intervention and control arms in the 3 years (2012, 2013 and 2014). Since there was no significant difference with regard to the variables of interest at baseline between the intervention and control arms, we attributed any observed difference in the number of deliveries to the intervention. Moreover, we believe that comparing the mean number of deliveries before and after the intervention in the same facilities accounted for any potential facility-level clustering (**see response to query 3 above**). In addition, we did not use linear regression because the aim of the study was not to identify the predictors of utilisation of delivery services. We believe the information we got and presented ($F(1,46)=18.85$, $p<0.001$) from ANOVA is as useful as the one we would have obtained from linear regression which looks at the goodness of fit for the model and the explained variance (R^2). Due to other technical issues, we could

not run analysis of covariance (ANCOVA) to account for the differences in gravidity. However, we have taken note of this and explained this weakness in our limitation section (**see page 14**)

Results

Query 19: Please include some analysis of participants lost to follow up. Are these women different from retained participants, and if so, how?

Response: We appreciate the comment; we have responded to this point already (**see response to query 4 above).**

Limitations:

Query 20: The authors should describe biases related to non-random allocation of facilities and whether there are potential selection biases related to including only public-sector facilities.

Response: We appreciate this guidance. We have described the biases related to non-random allocation of facilities (**see page 14**). We have explained that at the time of the study there were no private facilities providing obstetric and newborn healthcare services in the district (**see page 10**).

Minor comments:

Query 21: Line 84: Demographic & Health Survey should be capitalized

Response: This has been corrected accordingly (**see page 4**)

Query 22: Line 108: change aim to objective, remove “study also” for clarity

Response: This has been corrected accordingly (**see page 5**)

Query 23: Lines 252 and 254: please include units

Response: This has been corrected accordingly (**see page 11, 12**)

Reviewer #3:

This is a very important and informative study. However, I have a few concerns that need to be addressed.

Query 1: The statement "Participants in the intervention arm received ANC, health education and a mother-baby delivery pack when they arrived at the health facility for delivery" in your abstract is misleading. Please re-write.

Response: This has been corrected. The sentence now reads: In addition to the health education provided during routine ANC visits, participants in the intervention arm received health education and a mother-baby delivery pack when they arrived at the health facility for delivery (**see page 2**).

Query 2: Please re-write objectives stated in line 108-109 to align with the abstract and the rest of the document.

Response: The objective has been corrected (**see page 2**)

Query 3: The document still has a lot of typos; e.g line 326-importance versus important; 327 "community intervention trials are the only appropriate study design suited...". Please revisit and re-write for clarity.

Response: We have proof-read the whole document and corrected all the typo errors.

Query 4: You mention that study participants were identified from the first ANC visits, was this part of your screening criteria?

Response: Yes, the participants were identified during their first ANC visit so that they could be screened for their eligibility. Pregnant people who came for ANC at any gestation, regardless of the number of visits, was eligible to participate. However, since the outcome of interest was place of delivery (institutional or home delivery), care was taken to ensure that only women whose expected date of delivery (EDD) fell before 30th Nover, 2014 were recruited into the study (**see page 7**).

Query 5: You later state these differences in health service utilization, alongside other variables like gravidity, first ANC utilization, how did you take care of these and other obvious confounders? Please describe your multivariate analysis in more detail.

Response: We thank the reviewer for the insightful comments. We have explained above (under reviewer # 2 query 18) that the objective of the study was to assess the effect of provision of additional health education during antenatal care (ANC) and a non-financial incentive on institutional deliveries. In our view, this effect

can be tested using different models: analysis of various (ANOVA) or regression (linear, logistic or Poisson). In this case, we opted for one way ANOVA. We compared the mean number of deliveries between the intervention and control arms in the 3 years (2012, 2013 and 2014).

Since there was no significant difference with regard to the variables of interest at baseline between the intervention and control arms, we attributed any observed difference in the number of deliveries to the intervention. Moreover, we believe that comparing the mean number of deliveries before and after the intervention in the same facilities accounted for any potential facility-level clustering (**see response to query 3 above**). In addition, we did not use multivariate linear regression models because the objective of the study was not to identify the predictors of utilisation of delivery services. We believe the information we got and presented ($F(1,46)=18.85$, $p<0.001$) from ANOVA is as useful as the one we would have obtained from linear regression which looks at the goodness of fit for the model and the explained variance (R^2).

Query 6: Please report ALL secondary outcomes in the outcome tables and results section before embarking on their discussion.

Response: We appreciate the guidance by the reviewer. We have now reported all the secondary outcomes in the results section (**see page 13**)

Query 7: Line 257 states, "health facility deliveries in the intervention and control sites from January 2012 to Dec 2014....", and then proceed with the same narrative in line 182 and else where. Was baseline data collection part of this study? Please clarify confusion this because you state in line 319 that "the study was conducted over a one year period". Please also clarify how you treated baseline and comparative data in this/these studies.

Response: We thank the reviewer for the comment. We have now explained that this was a three-year study (2012 to 2014) analysing baseline delivery data for 2012 and 2013 followed by one-year (1st January to 31st December 2014) prospective community intervention trial conducted in Monze district, Zambia (**see page 6**).

Query 8: Your conclusion seems to "mute" other outcomes described and discussed in the study/effect of other intervention components aside from provision of a other-baby delivery pack. Please explain this. Remove references from the conclusion section.

Response: The conclusion has been edited both in the abstract and main document and highlights all the outcomes described and discussed in the document.

Query 9: Line 343, "study findings also show that the intervention in implementable". What do you mean?? The authors did not investigate implementation and do not document any implementation outcomes in this particular study. They even mention quite a number of limitations including a lack of costing, cost-effectiveness data among others, making it premature to make such assertions/conclusions. I would also consider the last six sentences of the conclusion to fall back into the discussion section, and authors to direct the readers on solid conclusions derived from the study data presented in this manuscript, plus make appropriate recommendations for further research/investigation.

Response: This section has been edited and the sentence, "*study findings also show that the intervention is implementable*" has been deleted. We have also made recommendations for future research/investigation (**see page 16**)

-
