# Supplementary material for: Effect of a mother-baby delivery pack on institutional deliveries: A community intervention trial to address maternal mortality in rural Zambia
Source: PLoS One. 2024 Mar 11;19(3):e0296001. doi: 10.1371/journal.pone.0296001 (PMC10927137; doi:10.1371/journal.pone.0296001)
Supplement: Attachment — Submitted filename: Response to reviewer comments.pdf [file pone.0296001.s008.pdf]

Response to reviewer comments

Reviewer # 2

Query 1: The authors have thoughtfully addressed many of the points I raised in my first review of this paper. I appreciate their careful consideration of these broad issue, which required a significant revision on their part. This version of the manuscript is greatly improved. Overall, this version was much easier to read, and I especially appreciated the expanded introduction and methods sections.

It might be underselling the importance of this trial to refer to the mother-baby pack as a non-financial incentive. This is a key cost-related barrier that the authors directly addressed and I would hate for this finding to get lost, which it somewhat does in the current abstract and first paragraph of the discussion, and the conclusion. The authors might add that the pack in both places that the pack includes essential delivery supplies, which would otherwise be an out-of-pocket expense. Adding a few sentences to make it clear that an aim of the trial is to test the effect of covering this potential expense in-kind that is a major barrier to utilizing facility delivery may increase the reach of these important findings

Response: We appreciate the complements from the reviewer and the concern about our referring to the mother-baby pack as a non-financial incentive. We have now corrected this both in the abstract and discussion. We have also corrected the title and conclusion sections accordingly. The aim now reads as: ***To test the effect of provision of additional health education during antenatal care (ANC) and a mother-baby delivery pack on institutional deliveries in Monze, Zambia.***

Abstract

Query 2: It might be helpful to note that the 5,000 women were identified as eligible or enrolled at baseline but were not exposed to the intervention; otherwise this raises questions about loss to follow up (this point was addressed in the manuscript and response to reviewers). I would also clarify here that the 12.4% who completed the study were not included in analysis because of incomplete data (otherwise it is unclear why they were not included.

Response: We thank the reviewer for this observation. We have made the correction accordingly (**page 2 line 31 to 34**)

Query 2: The secondary outcomes remain poorly defined. For example, does "ANC" mean obtained any ANC vs. initiated ANC in the first trimester vs. completed at least 4 ANC visits, or something else?
- If the authors are concerned about word limit in the abstract given these other suggestions, they could remove the statistical software as this is not typically necessary to report in the abstract (the main text is sufficient).

Response: We appreciate the comment by the reviewer. We have now explained that ANC, PNC and under-five service utilisation means the time of the first visit, number of visits completed according to the national guidelines (**see page 8**).

A detailed explanation about ANC and PNC services in the country is provided in the introduction section.

Introduction:

Query 3: If 33% of women are delivering at home, I would not say it is consistent to state "most women still give birth at home" (line 104). Please revise "most" (of course this is a not insubstantial proportion and I support the authors' efforts to increase facility delivery). It does seem that at study baseline a majority of women in the district had home deliveries but this is not supported by the ZDHS data. If the authors have access to the 2013 ZDHS, they might estimate the proportion of home deliveries in rural areas of the study region, which would be available in the dataset and might be >50%.

Response: We appreciate the guidance from the reviewer. We have corrected the sentence accordingly **(see page 5 lines 122 to 124).** We have also provided the statistics for the study district in the methods section under study setting **(see page 7 lines 180 to 181)**

Methods:

Query 4: Intervention: Could the authors clarify, is it that mother-baby packs were made available for free at all facilities in the intervention area, or that each woman was specifically given one (say, at her home) around the time of delivery? The mother-baby pack should be stated as part of the intervention in the first sentence of this paragraph.

Response: We have clarified that the mother-baby delivery packs were kept at the health facility; pregnant women only received them at the time of delivery if they went to deliver at the health facility. Information about the packs and their content was provided during the health education sessions as women went for their ANC visits (**see pages 8 and 9**). We have corrected the first sentence accordingly.

Query 5: Randomization: how was the actual randomization done, was it a coin flip or something else? Or did someone decide that west would be intervention?

Response: We appreciate the comment from the reviewer. We have now explained in detail how randomisation was done **(see page 6 and 7)**

Query 6: Outcomes: same comment as in the abstract, the secondary outcomes remain poorly defined. Please specify how each outcome is operationalized, e.g., "mother received a postnatal care visit within 48 hours of delivery." This is somewhat done in the results but should be here as well. If women are followed through their first PNC visit, does this mean that under 5 clinic utilization refers to PNC for the baby/newborn follow up? It might be helpful to be more explicit about this, as I assumed this was care for acute illnesses until I read the results section

Response: We have now explained how each secondary outcome was operationalised (**see page 9**). For PNC there were three measures: did the mother and her baby receive PNC? Time after delivery when PNC was received? Number of PNC visits. Similarly, for ANC, the measures were: did the pregnant woman use ANC? Gestation at ANC booking, and number of ANC visits completed.

Results:

Query 7: I appreciate that the authors included all p-values. However, while we might state that a p-value of .07 is not significant, it does suggest that there is an underlying difference that may be meaningful in some way or may be significant if the sample size was a little larger (line 299).

Response: We appreciate the observation by the reviewer. We have now corrected this sentence to read: "*There was a notable difference in the number of children between the respondents from the intervention (mean=3.6, SD=2.3) and control arms (mean=3.4, SD=2.2); however, the difference did not reach statistical significance (p=0.07)* (**see page 31**)

Discussion:

Query 8: Paragraph beginning on line 351: what were the non-financial incentives in the other studies referenced? It would be helpful to more directly compare what kinds of incentives they were.

Response: We appreciate the guidance from the reviewer. We have now described the incentives provided in the cited studies **(see page 15 lines 397 to 407)**

Query 9: This study does have strengths as the authors note on the paragraph starting on line 409. However, I think it is not reasonable to say that this was the only possible study design for this particular trial because in theory mother-baby packs and health education could be randomized at the individual level (I agree with the authors that theirs is an appropriate design). I would also remove the sentence starting on line 417; yes longer data collection periods may have challenges but that does not mean that the accuracy is always lower.

Response: We appreciate the concern from the reviewer. We have now deleted the sentence accordingly (**see page 17 lines 466 and 467**)

Minor comments:

Query 10: Line 158: “assent” instead of “ascent”

Response: We have corrected this and all other typo errors in the document

Reviewer #3:

Query 1: Description of secondary outcomes is still vague

Response: we appreciate the observation by the reviewer. We have now described the secondary outcomes in detail. We have explained how each secondary outcome was operationalised (**see page 9**). For PNC there were three measures: did the mother and her baby receive PNC? Time after delivery when PNC was received? Number of PNC visits. Similarly, for ANC, the measures were: did the pregnant woman use ANC? Gestation at ANC booking, and number of ANC visits completed.

Query 2: How did you choose the eight health centres?

Response: We appreciate the observation from the reviewer. We have now explained how the 8 health centres were selected (**see page 6 and 7**)

Query 3: You state that the questionaires were administered by the midwives who were trained as data collectors. Are these the same midwives running the labour ward, AND, PNC clinics? How did you deal with potential bias during data collection?

Response: We thank the reviewer for the observation. We have now explained that the midwives who collected the data were not involved in the service (ANC, delivery and PNC) provision. We have also mentioned that several measures were taken to ensure quality and avoid bias during the data collection process: data collectors were trained and worked under the supervision of the principla investigators and other research team members. In addition, midwives who participated in data collection were not involved in service provision (**see page 10**).

Query 4: At what point was PNC data collected?

Response: We thank the reviewer for the question. We mentioned under data collection section that PNC data was collected after delivery and when women came for PNC visits (**see page 10**)
